# Identification and Differentiation of *Verticillium* Species and *V. longisporum* Lineages by Simplex and Multiplex PCR Assays

**DOI:** 10.1371/journal.pone.0065990

**Published:** 2013-06-18

**Authors:** Patrik Inderbitzin, R. Michael Davis, Richard M. Bostock, Krishna V. Subbarao

**Affiliations:** Department of Plant Pathology, University of California Davis, Davis, California, United States of America; University of Ottawa, Canada

## Abstract

Accurate species identification is essential for effective plant disease management, but is challenging in fungi including *Verticillium* sensu stricto (*Ascomycota, Sordariomycetes, Plectosphaerellaceae*), a small genus of ten species that includes important plant pathogens. Here we present fifteen PCR assays for the identification of all recognized *Verticillium* species and the three lineages of the diploid hybrid *V. longisporum.* The assays were based on DNA sequence data from the ribosomal internal transcribed spacer region, and coding and non-coding regions of *actin*, *elongation factor 1-alpha*, *glyceraldehyde-3-phosphate dehydrogenase* and *tryptophan synthase* genes. The eleven single target (simplex) PCR assays resulted in amplicons of diagnostic size for *V. alfalfae*, *V. albo-atrum, V. dahliae* including *V. longisporum* lineage A1/D3, *V. isaacii*, *V. klebahnii*, *V. nonalfalfae*, *V. nubilum*, *V. tricorpus*, *V. zaregamsianum,* and Species A1 and Species D1, the two undescribed ancestors of *V. longisporum*. The four multiple target (multiplex) PCR assays simultaneously differentiated the species or lineages within the following four groups: *Verticillium albo-atrum*, *V. alfalfae* and *V. nonalfalfae*; *Verticillium dahliae* and *V. longisporum* lineages A1/D1, A1/D2 and A1/D3; *Verticillium dahliae* including *V. longisporum* lineage A1/D3, *V. isaacii*, *V. klebahnii* and *V. tricorpus*; *Verticillium isaacii*, *V. klebahnii* and *V. tricorpus*. Since *V. dahliae* is a parent of two of the three lineages of the diploid hybrid *V. longisporum*, no simplex PCR assay is able to differentiate *V. dahliae* from all *V. longisporum* lineages. PCR assays were tested with fungal DNA extracts from pure cultures, and were not evaluated for detection and quantification of *Verticillium* species from plant or soil samples. The DNA sequence alignments are provided and can be used for the design of additional primers.

## Introduction

*Verticillium* sensu stricto is a small group of agriculturally important, plant- associated fungi that cause Verticillium wilt, a type of vascular wilt that causes significant economic losses of numerous crops and ornamentals in many parts of the world [1], [2], [3]. Among the ten species currently recognized in *Verticillium* sensu stricto, [4], [5], *V. dahliae* is most widespread and most economically important [1], [6], [7], but *V. albo-atrum* [8], *V. alfalfae* [9], [10], *V. longisporum* [11], [12], *V. nonalfalfae* [13], [14], *V. tricorpus* [8], [15] and *V. zaregamsianum* [16] also cause significant losses, *V. nubilum* causes disease in pathogenicity tests [15], and both *V. isaacii* and *V. klebahnii* have been recovered from lettuce and artichoke, respectively [4], [17]. One of the characteristic features of *Verticillium* species is the formation of resting structures [4]. The resting structures of *V. dahliae* consist of clusters of thick-walled cells called microsclerotia, which remain viable in the soil for at least fourteen years [18]. Because as few as two microsclerotia per gram of soil result in plant infection and yield losses [19], knowledge about the abundance of microsclerotia and other resting structures in the soil is an important factor to consider for disease management. *Verticillium* species also differ in host range and pathogenicity [9], [15], [20], [21], [22]; thus, expedient detection, quantification and identification of *Verticillium* species has been the focus of extensive research efforts. These included the design of numerous PCR-based assays, targeting *V. albo-atrum* [23], [24], [25], [26], [27], [28], [29], [30], *V. dahliae* [24], [25], [26], [27], [28], [29], [30], [31], [32], [33], [34], [35], [36], [37], [38], [39], [40], [41], [42], *V. longisporum* [30], [38], [43], [44], and *V. tricorpus* [27], [28], [29], [38], [45], in a variety of substrates including alfalfa [23], oilseed rape [44], olive [41], pepper [31], potato [28], [29], [34], [45], [46], strawberry [32], soil [27], [30], [32], [35], [36], [37], [38], [39], [40], spinach seed [42], tomato [31], and herbaceous hosts in general [24], [25], [26], [27], [33].

**Figure 1:**
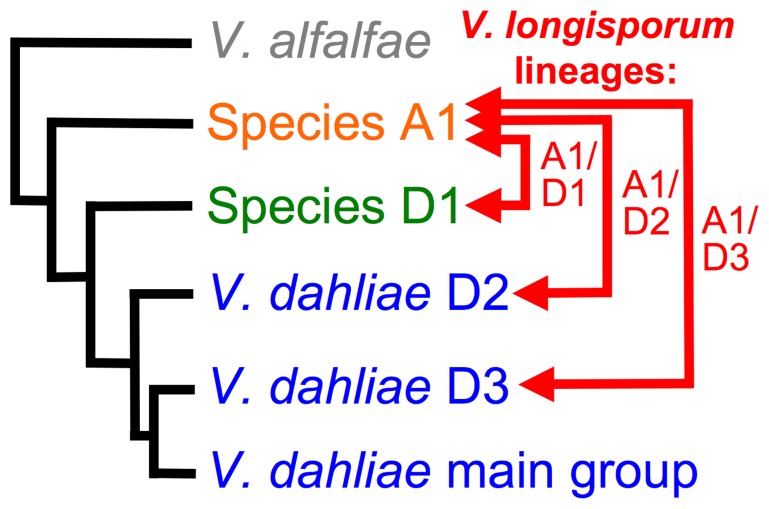
Evolutionary history of*Verticillium longisporum* illustrated by a cartoon phylogenetic tree based on Inderbitzin et al [**47**]. *Verticillium longisporum* evolved at least three different times by hybridization of Species A1with Species D1, *V. dahliae* lineage D2 and *V. dahliae* lineage D3, resulting in *V. longisporum* lineages A1/D1, A1/D2 and A1/D3, respectively. *Verticillium dahliae* isolates are in blue, Species D1 in green, Species A1 in orange and *V. alfalfae* in gray. Red arrows indicate parents of *V. longisporum,* connecting lines represent the three *V. longisporum* lineages. The *Verticillium dahliae* lineage D2 is marked as *‘V. dahliae* D2’ and comprises both *V. dahliae* isolates and D2-alleles of *V. longisporum* lineage A1/D2. The *Verticillium dahliae* lineage D3 (‘*V. dahliae* D3’) comprises only D3-alleles of *V. longisporum* lineage A1/D3. Most of the *V. dahliae* isolates in Inderbitzin et al. [47] belonged to the clade marked ‘*V. dahliae* main group’. Tree is not to scale.

Significant advances have recently been made in our understanding of the genetic diversity in *Verticillium.* Five new *Verticillium* species were described [4], including *V. isaacii* and *V. klebahnii* that are morphologically indistinguishable from *V. tricorpus,* and *V. alfalfae* and *V. nonalfalfae* that resemble *V. albo-atrum* and cannot be differentiated based on morphology. Also, the relationship of *V. dahliae* to the diploid hybrid *V. longisporum* was clarified [47]. It was found that *V. longisporum* consists of at least three groups that evolved independently by hybridization involving two unknown species and two lineages of *V. dahliae* (Figure 1). The two unknown species have never been found except as parents of *V. longisporum,* and have informally been named Species A1 and Species D1, and the two *V. dahliae* lineages are referred to as *V. dahliae* lineages D2 and D3. The three groups or lineages of *V. longisporum* evolved by independent hybridization of Species A1 with Species D1, *V. dahliae* lineage D2 and *V. dahliae* lineage D3, respectively, and accordingly, are referred to as *V. longisporum* lineage A1/D1, *V. longisporum* lineage A1/D2 and *V. longisporum* lineage A1/D3, respectively.

We used DNA sequence data generated in conjunction with phylogenetic and taxonomic studies of *Verticillium* [4], [47], and designed PCR assays for the identification of *Verticillium* species and *V. longisporum* lineages. The assays will be useful for diagnostics labs and research applications.

## Results and Discussion

**Figure 2:**
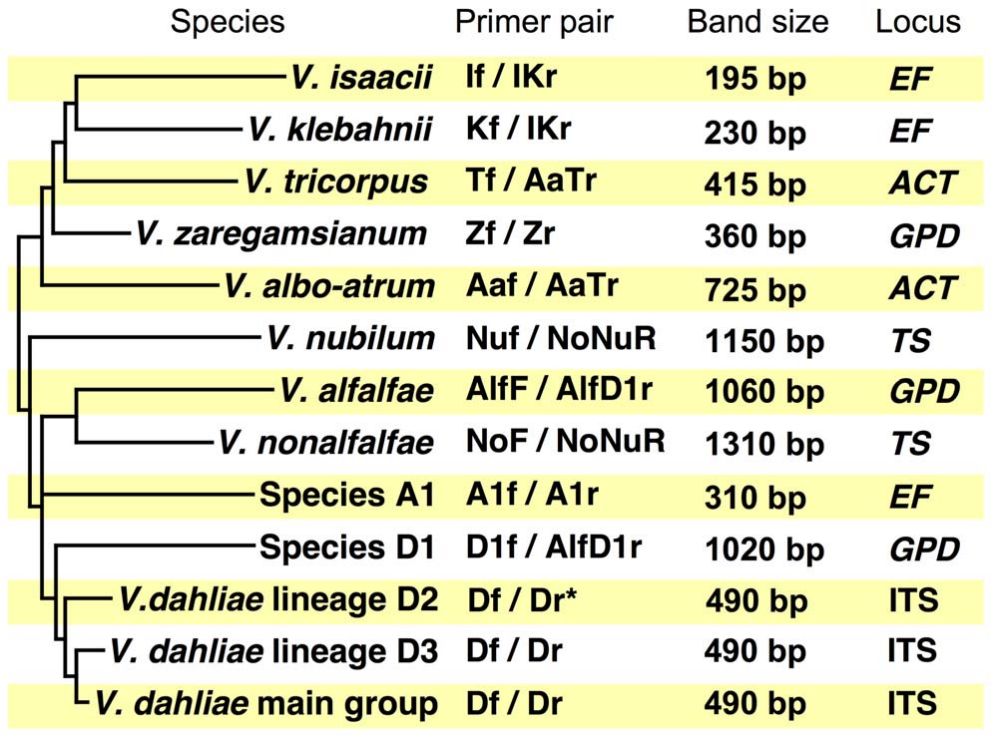
Specificity, expected band sizes and target loci of the eleven PCR primer pairs designed in this study. Phylogenetic tree on the left summarizes relationships of *Verticillium* species from Inderbitzin et al. [4], [47], branch lengths are not to scale. The asterisks indicates that primer pair Df/Dr only amplifies *V. dahliae* strains of *V. dahliae* lineage D2, but not strains of *V. longisporum* lineage A1/D2 (Figure 1). For details see text.

We designed eighteen PCR primers combined into eleven single-target (simplex) and four multi-target (multiplex) PCR assays for identification of all ten *Verticillium* species and *V. longisporum* lineages. PCR primer design was based on DNA sequence data of 257 *Verticillium* isolates at five loci, which were previously identified to species using type material [4], [47]. The targets of the eleven simplex PCR assays are shown in Figure 2, and included *V. albo-atrum, V. alfalfae, V. dahliae* including *V. longisporum* lineage A1/D3 (Figure 1), *V. isaacii, V. klebahnii, V. nonalfalfae, V. nubilum, V. tricorpus, V. zaregamsianum*, and Species A1 and Species D1, the two *V. longisporum* ancestors (Figure 1). The reliability of the primer pairs was confirmed in PCR assays as described below, and various combinations of primer pairs were evaluated for simultaneous amplification of more than one target species or *V. longisporum* lineage in multiplex PCR assays. Four multiplex PCR assays containing between five and seven primers were able to reliably amplify separate templates of the following morphologically or ecologically similar groups of species: *Verticillium albo-atrum, V. alfalfae* and *V. nonalfalfae*; *V. dahliae* including *V. longisporum* lineage A1/D3, *V. isaacii, V. klebahnii* and *V. tricorpus; V. dahliae* and *V. longisporum* lineages A1/D1, A1/D2 and A1/D3*; V. isaacii, V. klebahnii* and *V. tricorpus*.

### Isolate Sampling for Primer Design and PCR Assay Validation

DNA sequence data of many isolates of *Verticillium* are available in GenBank. However, since a large proportion of DNA sequences in GenBank is derived from isolates that are not correctly identified [48], [49], we designed the species and lineage-specific primers based on a set of 1290 DNA sequences from two taxonomic and phylogenetic studies of type specimens of *Verticillium* to guarantee correct identification [4], [47]. The sequences were from five loci of 257 *Verticillium* isolates and a *Gibellulopsis nigrescens* negative control. The largest proportion of isolates, 196 out of 257, was from *V. dahliae* and its close relative *V. longisporum.* The numbers for the other species ranged from four to fourteen as shown in Table 1. The DNA sequences retrieved for primer design represented the total genetic diversity at each of the five loci for all species, and were derived from one to eight isolates depending on the species (Table 1). The sequences were aligned separately for each locus, and PCR primers were designed as described in the Materials and Methods (Table 2). All alignments with primer sites are provided (Alignments S1, S2, S3, S4, S5).

**Table 1:** Numbers of isolates used for primer design in relation to numbers of isolates and genetic diversity of each*Verticillium* species in Inderbitzin et al. [4], [47].

Species/Totals	Isolates/species^A^	Isolates representing intraspecific diversity^B^	Isolates used for primer design^C^
*V. albo-atrum*	5	2	2
*V. alfalfae*	7	2	2
*V. dahliae*	154	8	8
*V. isaacii*	14	4	4
*V. klebahnii*	7	2	2
*V. longisporum*	42	3	7^D^
*V. nonalfalfae*	9	1	1
*V. nubilum*	4	1	1
*V. tricorpus*	5	3	3
*V. zaregamsianum*	10	4	4
**Total**	**257**	**30**	**34**

A Number of isolates in each species [4], [47].

B Number of isolates representing the genetic diversity across *ACT*, *EF*, *GPD*, ITS and *TS* [4], [47].

C Number of isolates used for primer design, for details see Table S4. Selected isolates represent the genetic diversity of each species at *ACT, EF, GPD,* ITS and *TS* [4], [47].

D The *V. longisporum* isolates with the best sequencing coverage were used at each locus (Table S4).

**Table 2:** Details of DNA sequence alignments used for primer design, including the numbers of taxa, alignment lengths and the numbers of primers designed at each locus.

Alignment locus	Number of taxa	Alignment length, characters	Number of primers designed
*ACT*	17	1203	3
*EF*	22	610	5
*GPD*	23	1221	5
ITS	17	619	2
*TS*	26	1464	3

**Figure 3:**
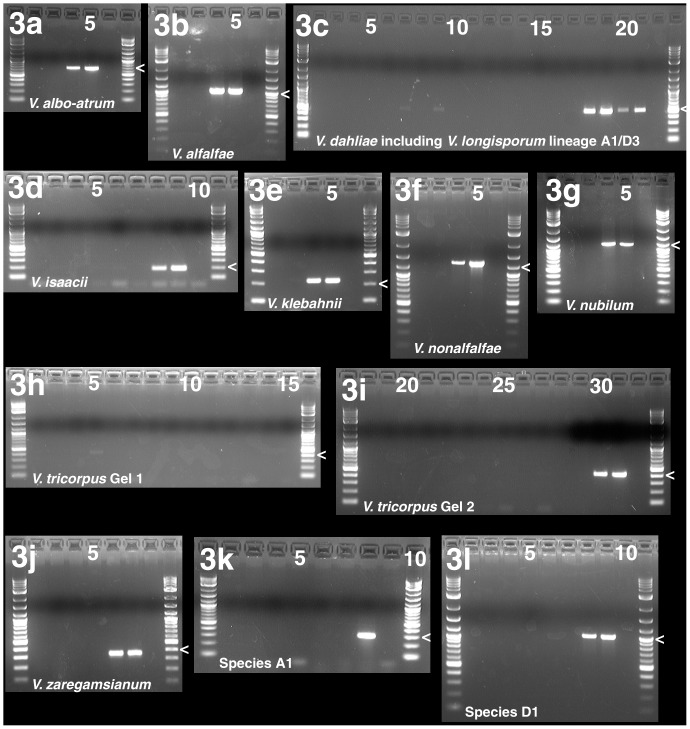
Simplex PCR assays are species-specific except for the*V. dahliae* simplex PCR assay that also amplifies *V. longisporum* lineage A1/D3. Agarose gels demonstrating selective amplification of all eleven species-specific simplex PCR assays. Each gel is delimited by 2-log ladders, penultimate lanes are negative controls except in Figure 3h, and relevant size markers are indicated by ‘<’. Lanes are numbered from left to right; numbers are given by the lanes for every fifth lane. The PCR assay target species are indicated at the bottom of gels. For explanation of isolates selected as negative controls see text. 3a. *Verticillium albo-atrum* PCR assay. Lanes 2, 3: *V. nubilum* strain PD621, 10 and 100 ng of DNA, respectively. Lanes 4, 5: *V. albo-atrum* strain PD693, 10 and 100 ng of DNA, respectively. Size marker = 700 bp. 3b. *Verticillium alfalfae* PCR assay. Lanes 2, 3: *V. nonalfalfae* strain PD592, 10 and 100 ng DNA. Lanes 4, 5: *V. alfalfae* strain PD683, 10 and 100 ng DNA. Size marker = 1000 bp. 3c. *Verticillium dahliae* PCR assay. Lanes 2, 3: *V. albo-atrum* strain PD670, 10 and 100 ng DNA. Lanes 4, 5: *V. alfalfae* strain PD338, 10 and 100 ng DNA. Lanes 6, 7: *V. klebahnii* strain PD347, 10 and 100 ng DNA. Lanes 8, 9: *V. nonalfalfae* strain PD592, 10 and 100 ng DNA. Lanes 10, 11: *V. nubilum* strain PD621, 10 and 100 ng DNA. Lanes 12, 13: *V. tricorpus* strain PD593, 10 and 100 ng DNA. Lanes 14, 15: *V. zaregamsianum* strain PD586, 10 and 100 ng DNA. Lanes 16, 17: *V. isaacii* strain PD341, 10 and 100 ng DNA. Lanes 18, 19: *V. dahliae* strain PD323, 10 and 100 ng DNA. Lanes 20, 21: *V. longisporum* lineage A1/D3 strain PD589, 10 and 100 ng DNA. Size marker = 500 bp. Note that the *V. dahliae* assay also amplifies *V. longisporum* lineage A1/D3, see lanes 20 and 21. 3d. *Verticillium isaacii* PCR assay. Lanes 2, 3: *V. klebahnii* strain PD347, 10 and 100 ng DNA. Lanes 4, 5: *V. klebahnii* strain PD407, 10 and 100 ng DNA. Lanes 6, 7: *V. tricorpus* strain PD593, 10 and 100 ng DNA. Lanes 8, 9: *V. isaacii* strain PD341, 10 and 100 ng DNA. Size marker = 200 bp. 3e. *Verticillium klebahnii* PCR assay. Lanes 2, 3: *V. isaacii* strain PD341, 10 and 100 ng DNA. Lanes 4, 5: *V. klebahnii* strain PD347, 10 and 100 ng DNA. Size marker = 200 bp. 3f. *Verticillium nonalfalfae* PCR assay. Lanes 2, 3: *V. alfalfae* strain PD683, 10 and 100 ng DNA. Lanes 4, 5: *V. nonalfalfae* strain PD592, 10 and 100 ng DNA. Size marker = 1200 bp. 3g. *Verticillium nubilum* PCR assay. Lanes 2, 3: *V. nonalfalfae* strain PD592, 10 and 100 ng DNA. Lanes 4, 5: *V. nubilum* strain PD741, 10 and 100 ng DNA. Size marker = 1200 bp. 3h, 3i. *Verticillium tricorpus* PCR assay. Lanes 2, 3: *V. dahliae* strain PD322, 10 and 100 ng DNA. Lanes 4, 5: *V. longisporum* lineage A1/D1 strain PD591, 10 and 100 ng DNA. Lanes 6, 7: *V. longisporum* lineage A1/D2 strain PD356, 10 and 100 ng DNA. Lanes 8, 9: *V. alfalfae* strain PD338, 10 and 100 ng DNA. Lanes 10, 11: *V. nonalfalfae* strain PD592, 10 and 100 ng DNA. Lanes 12, 13: *V. nubilum* strain PD621, 10 and 100 ng DNA. Lanes 14, 15: *V. albo-atrum* strain PD670, 10 and 100 ng DNA. Lanes 18, 19: *V. albo-atrum* strain PD693, 10 and 100 ng DNA. Lanes 20, 21: *V. zaregamsianum* strain PD586, 10 and 100 ng DNA. Lanes 22, 23: *V. zaregamsianum* strain PD739, 10 and 100 ng DNA. Lanes 24, 25: *V. isaacii* strain PD341, 10 and 100 ng DNA. Lanes 26, 27: *V. klebahnii* strain PD347, 10 and 100 ng DNA. Lanes 28, 29: *Gibellulopsis nigrescens* strain PD595, 10 and 100 ng DNA. Lanes 30, 31: *V. tricorpus* strain PD685, 10 and 100 ng DNA. Size marker = 400 bp. 3j. *Verticillium zaregamsianum* PCR assay. Lanes 2, 3: *V. tricorpus* strain PD685, 10 and 100 ng DNA. Lanes 4, 5: *V. tricorpus* strain PD703, 10 and 100 ng DNA. Lanes 6, 7: *V. zaregamsianum* strain PD586, 10 and 100 ng DNA. Size marker = 400 bp. 3k. Species A1 PCR assay. Lanes 2, 3: *V. dahliae* strain PD323, 10 and 100 ng DNA. Lanes 4, 5: *V. dahliae* strain PD327, 10 and 100 ng DNA. Lanes 6, 7: *V. dahliae* strain PD332, 10 and 100 ng DNA. Lane 8: *V. longisporum* lineage A1/D1 strain PD720, 10 ng DNA. Size marker = 300 bp. 3l. Species D1 PCR assay. Lanes 2, 3: *V. dahliae* strain PD328, 10 and 100 ng DNA. Lanes 4, 5: *V. longisporum* lineage A1/D2 strain PD402, 10 and 100 ng DNA. Lanes 6, 7: *V. longisporum* lineage A1/D3 strain PD687, 10 and 100 ng DNA. Lanes 8, 9: *V. longisporum* lineage A1/D1 strain PD640, 10 and 100 ng DNA. Size marker = 1000 bp.

The isolates employed for the validation of the PCR assays included *Verticillium* and *Gibellulopsis* strains used for primer design [4], [47], in addition to a *Musicillium theobromae* negative control, twelve uncharacterized isolates of *V. dahliae, V. isaacii* and *V. klebahnii,* and *V. longisporum*. Primer pairs were first evaluated in simplex PCR assays using different numbers of isolates as positive and negative controls for each primer pair as shown in Table 3, depending on the genetic diversity of the target species, the numbers of isolates available, and the numbers of non-target species with similar primer sites as described in the Materials and Methods. The PCR conditions are detailed in Table 4. The PCR banding patterns for the simplex assays are shown in Figure 3, and the results from PCRs with additional isolates are in Figure S1 and Figure S2.

**Table 3:** Isolates used in the validation of the*Verticillium* simplex PCR assays.

Simplex assay target species	Target isolates^A^	Negative controls^B^
*V. albo-atrum*	*V. albo-atrum* strains PD693, PD746, PD747, PD748	*V. nubilum* strain PD621
*V. alfalfae*	*V. alfalfae* strains PD338, PD353, PD489, PD620, PD681, PD682, PD683	*V. nonalfalfae* strain PD592
*V. dahliae* and *V. longisporum*lineage A1/D3	*V. dahliae* strains PD323, PD328, PD331, PD615, PD656,PD718; *V. longisporum* lineage A1/D3 strain PD589	*V. albo-atrum* strain PD670; *V. alfalfae* strain PD338; *V. isaacii* strain PD341; *V. klebahnii* strain PD347; *V. nonalfalfae* strain PD592; *V. nubilum* strain PD621; *V. tricorpus* strain PD593; *V. zaregamsianum* strain PD586
*V. isaacii*	*V. isaacii* strain PD341	*V. klebahnii* strain PD347, PD407; *V. tricorpus* strain PD593
*V. klebahnii*	*V. klebahnii* strain PD347	*V. isaacii* strain PD341
*V. nonalfalfae*	*V. nonalfalfae* strains PD592, PD616, PD626, PD744,PD745, PD808, P809, PD810, PD811	*V. alfalfae* strain PD683
*V. nubilum* C	*V. nubilum* strains PD621, PD702, PD741, PD742	*V. nonalfalfae* strain PD592
*V. tricorpus*	*V. tricorpus* strain PD685	*V. albo-atrum* strain PD670, PD693; *V. alfalfae* strain PD338; *V. dahliae* strain PD322; *V. isaacii* strain PD341; *V. klebahnii* strain PD347; *V. longisporum* lineage A1/D1 strain PD591; *V. longisporum* lineage A1/D2 strain PD356; *V. nonalfalfae* strain PD592; *V. nubilum* strain PD621; *V. zaregamsianum* strains PD586, PD739; *Gibellulopsis nigrescens* strain PD595
*V. zaregamsianum* C	*V. zaregamsianum* strain PD586, PD733, PD736, PD737,PD738, PD740	*V. tricorpus* strains PD685, PD703
Species A1	*V. longisporum* lineage A1/D1 strain PD720	*V. dahliae* strain PD323, PD327, PD332
Species D1	*V. longisporum* lineage A1/D1 strain PD640	*V. dahliae* strain PD328; *V. longisporum* lineage A1/D2 strain PD402; *V. longisporum* lineage A1/D3 strain PD687

A Target isolates served as positive controls for the respective species and lineage specific primer pairs. Strain numbers were compiled from Figure 3 and Figure S2.

B Negative control isolates were selected to represent all species that differed from the respective target species by four or fewer substitutions at the more divergent primer site, or were randomly selected if all non-target species differed by more than four substitutions from the target species (Table S4). Strain numbers were compiled from Figure 3 and Figure S2.

C PCR primer pair targeting this species was not used in any multiplex PCR assay and was thus also tested with the set of 26 isolates in Table 5 used for multiplex assay validation.

**Table 4:** Details of*Verticillium* simplex PCR assays, including target loci, primer pairs, DNA template concentrations, PCR annealing temperatures, numbers of PCR cycles, PCR product sizes, and agarose gel concentrations for gel electrophoresis.

Target species	Target locus	Primer pair^A^	DNA template/reaction^A^, ng	Annealing temperature^B^, °C	PCR cycles^B^	PCR amplicon, bp	Agarose in TAE buffer, % (w/v)
*V. albo-atrum*	*ACT*	AaF/AaTr	10 or 100	62	35	725	1.5
*V. alfalfae*	*GPD*	AlfF/AlfD1r	10 or 100	62	35	1060	1.5
*V. dahliae* C	*ITS*	Df/Dr	1 or 10	67	32	490	1.5 or 2
*V. isaacii*	*EF*	If/IKr	10 or 100	64	35	195	2
*V. klebahnii*	*EF*	Kf/IKr	10 or 100	62	35	230	2
*V. nonalfalfae*	*TS*	NoF/NoNuR	10 or 100	64	35	1310	1.5
*V. nubilum*	*TS*	Nuf/NoNuR	10 or 100	62	35	1150	1.5
*V. tricorpus*	*ACT*	Tf/AaTr	10 or 100	64	35	415	1.5 or 2
*V. zaregamsianum*	*GPD*	Zf/Zr	10 or 100	64	35	360	2
Species A1	*EF*	A1f/A1r	10 or 100	64	35	310	2
Species D1	*GPD*	D1f/AlfD1r	10 or 100	70	35	1020	1.5

A Each 25 µl PCR reaction contained the following: 1.25 µl of each primer from 10 µM stocks, 12.5 µl Promega master mix, and 10 µl template containing 1, 10 or 100 ng DNA.

B The PCR program consisted of a 2 min initial denaturation step at 94°C, 32 or 35 cycles of 10 sec at 94°C, 20 sec at the PCR assay-dependent annealing temperature, and 1 min at 72°C, followed by a final extension of 7 min at 72°C. PCR reactions were set up at room temperature under sterile conditions and run immediately, or were stored in a freezer.

C Assay does not differentiate *V. dahliae* from *V. longisporum* lineage A1/D3.

**Figure 4:**
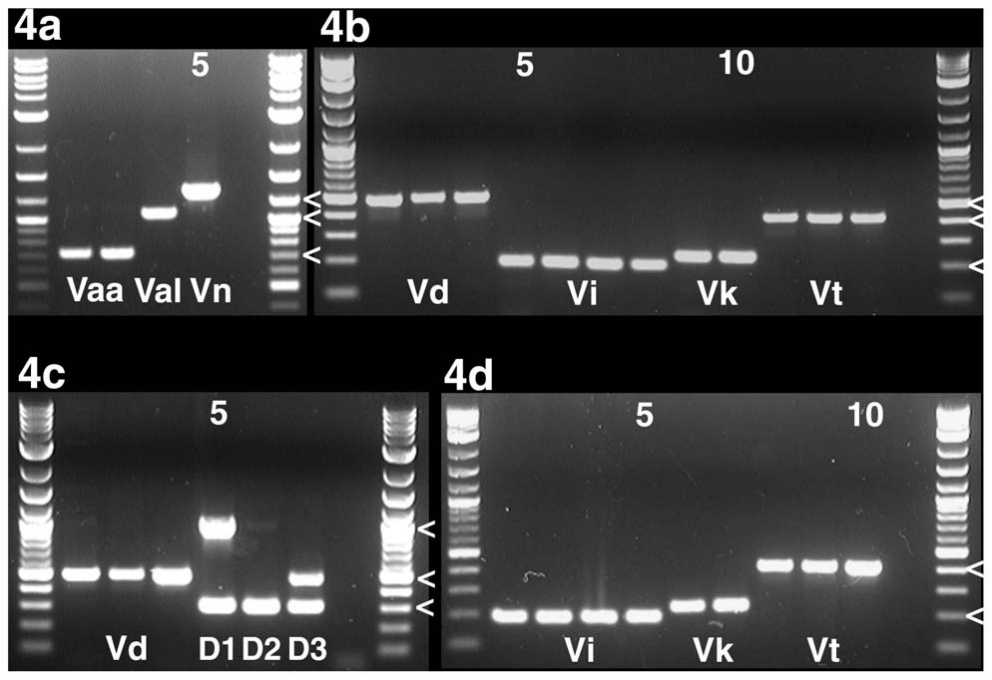
Multiplex PCR assays identify genetically diverse target isolates. Each agarose gel displays the results of one of the four multiplex PCR assays, controls with none-target isolates are shown in Figure S3. Gels are delimited by 2-log ladders, penultimate wells are negative controls, and relevant size markers are indicated by ‘<’. Lanes are numbered from left to right; numbers are given for every fifth lane. Abbreviations below bands indicate species and *V. longisporum* lineages as given for each part figure below. All lanes contain 100 ng template DNA. For an explanation of isolates included see text. 4a. *Verticillium albo-atrum* – *V. alfalfae* – *V. nonalfalfae* multiplex PCR assay. Lanes 2, 3: *V. albo-atrum* strains PD670, PD693. Lane 4: *V. alfalfae* strain PD338. Lane 5: *V. nonalfalfae* strain PD592. Size markers = 700 bp, 1000 bp, 1200 bp. 4b. *Verticillium dahliae – V. isaacii – V. klebahnii – V. tricorpus* multiplex PCR assay. Lanes 2–4: *V. dahliae* strains PD322, PD327, PD502. Lanes 5–8: *V. isaacii* strains PD341, PD343, PD618, PD752. Lanes 9, 10: *V. klebahnii* strains PD347, PD407. Lanes 11–13: *V. tricorpus* strains PD593, PD685, PD703. Size markers = 200 bp, 400 bp, 500 bp. 4c. *Verticillium dahliae – V. longisporum* PCR multiplex PCR assay. Lanes 2–4: *V. dahliae* strains PD322, PD327, PD502. Lane 5: *V. longisporum* lineage A1/D1 strain PD348. Lane 6: *V. longisporum* lineage A1/D2 strain PD356. Lane 7: *V. longisporum* lineage A1/D3 strain PD589. Size marker = 300 bp, 500 bp, 1000 bp. 4d. *Verticillium isaacii – V. klebahnii – V. tricorpus* multiplex PCR assay. Lanes 2–5: *V. isaacii* strains PD341, PD343, PD618, PD752. Lanes 6, 7: *V. klebahnii* strains PD347, PD407. Lanes 8–10: *V. tricorpus* strains PD593, PD685, PD703. Size markers = 200 bp, 400 bp.

All multiplex PCR assays were validated with a set of 26 representative isolates (Table 5). The PCR conditions are described in Table 6 and the PCR banding patterns for the multiplex assays are shown in Figure 4, and in Figures S2 and S3 for additional isolates. The total number of isolates involved in the validation of each primer pair in both simplex and multiplex PCR assays varied from 27 to 43 as summarized in Table S1.

**Table 5:** *Verticillium, Gibellulopsis* and *Musicillium* isolates used to validate the multiplex PCR assays, for details on isolate selection see Materials and Methods.

Species	Strain identifier^A^
*G. nigrescens*	PD710
*M. theobromae*	PD686
*V. albo-atrum*	PD670
*V. albo-atrum*	PD693
*V. alfalfae*	PD338^B^
*V. dahliae*	PD322^C^
*V. dahliae*	PD327
*V. dahliae*	PD502
*V. isaacii*	PD341
*V. isaacii*	PD343
*V. isaacii*	PD618
*V. isaacii*	PD752
*V. klebahnii*	PD347
*V. klebahnii*	PD407
*V. longisporum* lineage A1/D1	PD348^D^
*V. longisporum* lineage A1/D2	PD356
*V. longisporum* lineage A1/D3	PD589
*V. nonalfalfae*	PD592
*V. nubilum*	PD621^E^
*V. tricorpus*	PD593
*V. tricorpus*	PD685
*V. tricorpus*	PD703
*V. zaregamsianum*	PD586
*V. zaregamsianum*	PD731
*V. zaregamsianum*	PD735
*V. zaregamsianum*	PD739

A See Inderbitzin et al. [4], [47] for *Verticillium* and *Gibellulopsis* strain information, for information on the *M. theobromae* strain see text.

B Replaced at times by genetically equivalent *V. alfalfae* strain PD683 [4].

C Replaced at times by genetically equivalent *V. dahliae* strains PD328, PD323 and PD332 [47].

D Replaced at times by genetically equivalent *V. longisporum* lineage A1/D1 strains PD591, PD640, PD720 [47].

E Replaced at times by genetically equivalent *V. nubilum* strain PD741 [4].

**Table 6:** Details of *Verticillium* multiplex PCR assays, including target loci, primer pairs, DNA template concentrations, PCR annealing temperatures, numbers of PCR cycles, PCR product sizes, and agarose gel concentrations for gel electrophoresis.

Target species	Target loci	Primer pairs^A^	DNA template/reaction^A^, ng	Annealingtemperature^B^, °C	PCR cycles^B^	PCR amplicon, bp	Agarose in TAE buffer, % (w/v)
*V. albo-atrum – V. alfalfae – V. nonalfalfae*	*ACT, GPD, TS*	AlfF/AlfD1r, NoF/NoNuR, AaF/AaTr	10 or 100	64	35	725, 1060, 1310	1.5
*V. dahliae – V. longisporum lineages*	*GPD, EF, ITS*	D1f/AlfD1r, A1f/A1r, Df/Dr	10 or 100	64	35	490, 310, 1020	1.5
*V. dahliae* C *– V. isaacii – V. klebahnii – V. tricorpus*	*ITS, EF, ACT*	Df/Dr, If/IKr, Kf/IKr, Tf/AaTr	10 or 100	64	35	490, 195, 230, 415	2
*V. isaacii – V. klebahnii – V. tricorpus*	*EF, ACT*	If/IKr, Kf/IKr, Tf/AaTr	10 or 100	62	35	195, 230, 415	2

A Each 25 µl PCR reaction contained the following: 2.5 µl primer stock (see Tables 7, 8, 9, 10), 12.5 µl Promega master mix, and 10 µl template containing 10 or 100 ng DNA.

B The PCR program consisted of a 2 min initial denaturation step at 94°C, 32 or 35 cycles of 10 sec at 94°C, 20 sec at the PCR assay-dependent annealing temperature, and 1 min at 72°C, followed by a final extension of 7 min at 72°C. PCR reactions were set up at room temperature under sterile conditions and run immediately, or were stored in a freezer.

C Assay does not differentiate *V. dahliae* from *V. longisporum* lineage A1/D3.

### Identifying *Verticillium* Species and Setting Up PCR Assays

The deployment of the PCR assays described here assumes identification of *Verticillium* at the genus level using morphological characters, which can be challenging. If a fungal culture isolated from an agricultural substrate contains thick-walled, dark-pigmented resting structures, and long, narrow conidiogenous cells arranged in whorls along the main axis of the conidiophore, chances are high that it is *Verticillium* [4]. However, there are exceptions. *Gibellulopsis nigrescens* and *Musicillium theobromae* are associated with plants, resemble *Verticillium* species in terms of conidiophore and resting structure morphology, but are phylogenetically distinct and belong to different genera [5]. Also, numerous other unrelated fungi have conidiophores suggestive of *Verticillium* [50], [51], [52]. Should all the PCR assays fail and positive control isolates are not available, confirmation of genus identity can be performed by sequencing the ITS region and undertaking a nucleotide BLAST search at GenBank, or preferably, phylogenetic analyses with a *Verticillium* ITS dataset that contains ex-type sequences [4], available from TreeBASE at www.treebase.org [53].

To help select the most appropriate PCR assay to use, a morphology-based key is available for preliminary identification to species or species groups [4]. For identification of *V. dahliae* and *V. longisporum,* the *V. dahliae – V. longisporum* multiplex assay should generally be used, because it is the only one of the assays presented here that is able to distinguish *V. dahliae* from all *V. longisporum* lineages. The *V. dahliae* simplex assay in Table 4 also amplifies isolates of the *V. longisporum* lineage A1/D3 (Figure 3), and can thus lead to false positive results.

*Verticillium dahliae* and *V. longisporum* are the most difficult *Verticillium* species to identify by PCR assay, because *V. dahliae* is the parent of two of the three *V. longisporum* lineages, *V. longisporum* lineage A1/D2 and *V. longisporum* lineage A1/D3 (Figure 1). Due to the high genetic similarity between *V. longisporum* and *V. dahliae,* PCR primers specific to *V. dahliae* protein-coding genes will in most cases amplify the orthologs in *V. longisporum* lineages A1/D2 and A1/D3. Our multiplex PCR assay differentiates *V. dahliae* from *V. longisporum* by targeting the Species A1 *EF* allele that is unique to *V. longisporum*. *Verticillium longisporum* lineage A1/D1 is differentiated from the other lineages by an amplicon of the Species D1 *GPD* allele, and *V. longisporum* lineage A1/D3 is the only lineage that has an ITS region derived from *V. dahliae*. The other two lineages’ ITS regions are from Species A1. Due to concerted evolution, all of the *V. longisporum* lineages appear to have just one type of ITS region [47]. Thus, the *V. longisporum* lineage A1/D1 PCR banding pattern consists of the 310-bp Species A1 *EF* and the 1020-bp Species D1 *GPD* amplicons, the *V. longisporum* lineage A1/D2 banding pattern consists of the 310-bp Species A1 *EF* amplicon, and the *V. longisporum* lineage A1/D3 pattern consists of the 310-bp Species A1 *EF* and the 490-bp ITS *V. dahliae* amplicons (Figure 2, Figure 4). The *V. longisporum* lineage A1/D2 banding pattern is identical to the pattern expected for Species A1. However, Species A1 has never been found and is only known as one of the parents of *V. longisporum* [47].

**Figure 5:**
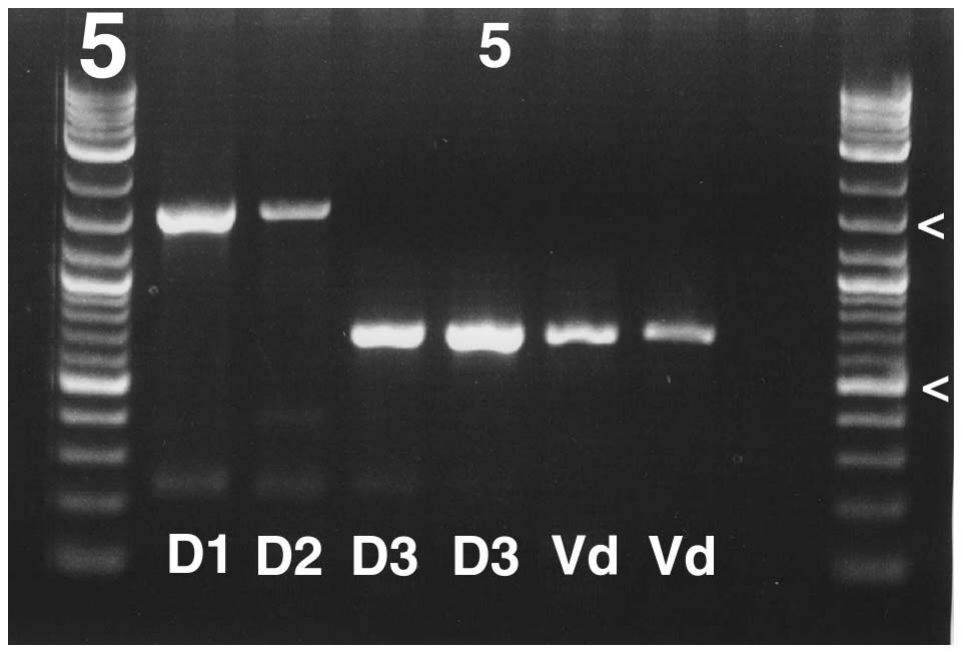
*Verticillium longisporum* lineage A1/D3 lacks a 839-bp SSU rRNA intron that is present in the other *V. longisporum* lineages. Agarose gel of Bas3/NS6 amplicons. Gel is delimited by 2-log ladders, penultimate lane is negative control, and relevant size markers are indicated by ‘<’ and correspond to 500 bp and 1500 bp, respectively. Lanes are numbered from left to right; fifth lane is numbered. Abbreviations below bands refer to *V. longisporum* lineages and *V. dahliae*. For information on isolates selected see text. Lane 2: *V. longisporum* lineage A1/D1 strain PD590. Lane 3: *V. longisporum* lineage A1/D2 strain PD730. Lane 4: *V. longisporum* lineage A1/D3 strain PD614. Lane 5: *V. longisporum* lineage A1/D3 strain PD715. Lane 6: *V. dahliae* strain Ls.1875. Lane 7: *V. dahliae* strain PD362.

PCR assays for the identification of *V. dahliae* and *V. longisporum* have previously been published, including the assay by Karapapa and Typas [43] who used the presence of a 839-bp intron in the nuclear SSU rRNA gene as a marker for *V. longisporum.* In agreement with the ITS data, *V. longisporum* lineage A1/D3 also lacks the SSU intron (Figure 5), and based on Karapapa and Typas’ assay, *V. longisporum* lineage A1/D3 would thus be identified as *V. dahliae*. A similar problem exists for *V. dahliae* diagnostic assays that target the ITS region [40], or protein coding genes which might falsely identify *V. longisporum* lineages A1/D2 and A1/D3 as *V. dahliae* [42]. However, *V. dahliae* and *V. longisporum* tend to have different host ranges, and in many cases, a *V. dahliae* assay that excludes all *V. longisporum* lineages may not be necessary.

Details of all fifteen PCR assays designed in this study for identification and differentiation of *Verticillium* species, including primers and other PCR conditions, are listed in Table 4 and Table 6. The setup of the multiplex PCR assays involves preparation of a primer master mix from 100 µM primer stocks as detailed in Tables 7, 8, 9, 10.

**Table 7:** Preparation of 125 µl primer stock for *V. albo-atrum–V. alfalfae–V. nonalfalfae* multiplex PCR assay sufficient for 50 25-µl PCR reactions.

Component	Volume, µl^A^
AlfF	6.25
NoF	6.25
NoNuR	6.25
AaF	6.25
AaTr	6.25
AlfD1r	6.25
diH20	87.5

A Primer initial concentrations = 100 µM each.

**Table 8:** Preparation of 125 µl primer stock for *V. dahliae–V. isaacii–V. klebahnii–V. tricorpus* multiplex PCR assay sufficient for 50 25-µl PCR reactions.

Component	Volume, µl^A^
AaTr	6.25
If	6.25
Kf	6.25
Tf	6.25
IKr	6.25
Df	3.125
Dr	3.125
diH20	87.5

A Primer initial concentrations = 100 µM each.

**Table 9:** Preparation of 125 µl primer stock for *V. dahliae–V. longisporum* lineages multiplex PCR assay sufficient for 50 25-µl PCR reactions.

Component	Volume, µl^A^
D1f	6.25
AlfD1r	6.25
A1f	6.25
A1r	6.25
Df	3.125
Dr	3.125
diH20	93.75

A Primer initial concentrations = 100 µM each.

**Table 10:** Preparation of 125 µl primer stock for *V. isaacii–V. klebahnii–V. tricorpus* multiplex PCR assay sufficient for 50 25-µl PCR reactions.

Component	Volume, µl^A^
AaTr	6.25
If	6.25
Kf	6.25
Tf	6.25
IKr	6.25
diH20	93.75

A Primer initial concentrations = 100 µM each.

### Screening for the Unknown *V. longisporum* Parents Species A1 and Species D1

The two informally named Species A1 and Species D1 [47] have never been found and are only known as parents of *V. longisporum* (Figure 1). Since neither the morphology nor ecology of Species A1 and Species D1 is known, and the two species may resemble *V. longisporum* and *V. dahliae* morphologically, PCR assays provide an opportunity to screen existing or new collections for isolates of Species A1 and Species D1. With the *V. dahliae – V. longisporum* multiplex PCR assay (Table 6), the Species D1-diagnostic PCR banding pattern is expected to consist of only the 1020-bp Species D1 band, and the Species A1 banding pattern would be identical to the banding pattern of *V. longisporum* lineage A1/D2 that comprises one 310-bp Species A1 band (Figure 2, Figure 4). To differentiate Species A1 from *Verticillium longisporum* lineage A1/D2, PCR reactions targeting protein coding genes, for instance with primer pairs VActF/VActR for *ACT*, VEFf/VEFr for *EF*, VGPDf2/VGPDr for *GPD* [47], would result in single amplicons that could be sequenced directly without cloning in Species A1, and in phylogenetic analyses would cluster with Species A1. Alternatively, primer pairs specific to allele D2 of *V. longisporum* lineage A1/D2 could be used to confirm the absence of allele D2. The primer pairs include ActF2d2/VActR targeting 503 bp of *ACT,* MATdf/MATdr targeting 419 bp of *MAT,* OxFd2/VOxR targeting 505 bp of *OX,* and TsFd2/VTs2R targeting 511 bp of *TS* [47].

## Materials and Methods

### DNA Sequence Data

A total of 104 DNA sequences from ten *Verticillium* species and *Gibellulopsis nigrescens* were retrieved from GenBank or the Broad Institute website (Table S2). Ninety-five of the *Verticillium* sequences were from 34 isolates that represented the genetic diversity at five loci, including the ribosomal internal transcribed spacer (ITS) region, *actin* (*ACT*), *elongation factor 1-alpha* (*EF*), *glyceraldehyde-3-phosphate dehydrogenase* (*GPD*), and *tryptophan synthase* (*TS*), in the ten *Verticillium* species in Inderbitzin et al. [4], [47]. Four *Verticillium* sequences from Klosterman et al. [54] and Pramateftaki et al. [55] provided DNA sequencing coverage for non-specific primers outside of the regions sequenced by Inderbitzin et al. [4], [47].

The ITS sequence of *Musicillium theobromae* strain PD686 (CBS 110322), an additional negative control [5], was generated using primers ITS1-F [56] and ITS5 [57] with settings described in Inderbitzin et al. [47]. The ITS sequence was submitted to GenBank as JQ621980. The species identification of *M. theobromae* strain PD686 was based on GenBank ITS BLAST hits [58], and is thus tentative.

### DNA Sequence Alignments and Primer Design

DNA sequences were aligned separately for each locus (Alignments S1, S2, S3, S4 and S5) using CLUSTAL X version 2.0 [59], [60], and eighteen primers were designed manually in Geneious Pro version 4.8.5 [61] (Table S3). Primer specificity was achieved by maximizing the number of mismatches between a primer’s 3′-end and homologous sites in non-target lineages [62]. Primers were evaluated with OligoCalc [63], available at http://basic.northwestern.edu/biotools/OligoCalc.html (last accessed February 3, 2012) using default settings. Primer annealing temperatures were between 53°C and 58°C as determined by the Nearest Neighbor Method [63] (Table S3). Primer names were chosen to reflect primer specificity. For instance, forward primer ‘If’, named after *V. isaacii,* was used only for amplification of *V. isaacii*, whereas reverse primer ‘IKr’ that was named after *V. isaacii* and *V. klebahnii,* was part of both *V. isaacii* and *V. klebahnii*-specific primer pairs (Figure 2).

### Fungal Isolates, Cultures, DNA Extraction, PCR and Gel Electrophoresis

For information on all fungal isolates used in this study, see Inderbitzin et al. [4], [47], except for *V. dahliae* strains Ls.1867, Ls.1870, Ls.1871, Ls.1875, Ls.1877, Ls.1878, *V. isaacii* strains Ls.1864, Ls.1868, Ls.1869, and *V. klebahnii* strains Ls.1865 and Ls.1886, all isolated from lettuce in the Salinas area, and *V. longisporum* lineage A1/D1 strain PD725 ( = strain Vd13) from oilseed rape in Sweden [44], all retrieved from the Subbarao lab collection. All fungal isolates were derived from single conidia [4]. Culture conditions and DNA extraction protocols were as in Inderbitzin et al. [47]. PCRs were performed using GoTaq Colorless Master Mix (Promega Corp., Madison, WI, USA) in GeneMate 0.2 ml 8-strip PCR tubes (BioExpress, Kaysville, UT). Each PCR reaction comprised 10 µl template dilution containing 1, 10, or 100 ng DNA, 2.5 µl primer mixture (0.5 µM for each primer, except primers D3f and D3r that were 0.25 µM each when multiplexed) and 12.5 µl master mix, for a total volume of 25 µl. The PCR program consisted of a 2 min initial denaturation step at 94°C, 32 or 35 cycles of 10 sec at 94°C, 20 sec at the PCR assay-dependent annealing temperature, and 1 min at 72°C, followed by a final extension of 7 min at 72°C. PCR reactions were set up at room temperature under sterile conditions in a laminar flow hood wearing gloves and using plugged pipet tips to minimize contamination. The reactions were run immediately, or were stored in a freezer. PCR machines used were a GeneAmp PCR System 9700 (Applied Biosystems, Carlsbad, CA), a 2720 Thermal Cycler (Applied Biosystems, Carlsbad, CA) and a PTC-200 DNA Engine (BioRad Laboratories, Inc., Hercules, CA).

Agarose gel electrophoresis was performed in a RAGE RGX-60 gel box with a 20-sample comb (Cascade Biologics, Inc., Portland, Oregon) or a larger Bio-Rad Wide Mini Sub Cell gel box (Bio-Rad Life Science, Hercules, CA) with a 30-sample box. Gels were run between 30 to 70 minutes at 70–90 V, using various agarose concentrations (Table 4, Table 6). PCR product, 4–6 µl was loaded per well. A 2-log DNA ladder, 0.75 µg (New England Biolabs, Inc., Ipswich, MA) was loaded per well. Loading buffer contained xylene cyanol or bromophenol blue for small and large amplicons, respectively [64].

### Validation of PCR Assays

Each PCR primer pair (Table 4) was initially validated in a simplex PCR assay that included one representative of the target species as a positive control, and negative controls that consisted of one representative of each species that differed from the target species by four or fewer substitutions at the more variable primer site (Figure 3, Table S4). Further validation was performed with additional target and non-target isolates (Figure S2). Each PCR primer pair was tested in at least three different PCR runs, except for the Species A1, Species D1 and *V. dahliae* primer pairs.

Validation of multiplex PCR assays (Table 6) included two control isolates *Gibellulopsis nigrescens* strain PD595 [47] and the more distantly related *Musicillium theobromae* strain PD686 [5], and 24 *Verticillium* isolates representing the allelic diversity at *ACT, EF, GPD,* ITS and *TS* as determined by Inderbitzin et al. [4], [47] (Figure 4, Figure S3). Three strains represented *V. dahliae* lineages D2, D3 and the main group of *V. dahliae* [47], and one *V. alfalfae* isolate was included. Each multiplex PCR assay was run on three different PCR machines.

The *V. dahliae – V. isaacii – V. klebahnii – V. tricorpus* multiplex PCR assay was also validated with eleven genetically uncharacterized isolates from lettuce in California. These were *V. dahliae* strains Ls.1867, Ls.1870, Ls.1871, Ls.1875, Ls.1877, Ls.1878, *V. isaacii* strains Ls.1864, Ls.1868, Ls.1869, and *V. klebahnii* strains Ls.1865 and Ls.1886 obtained from the Subbarao lab collection. The PCR results were confirmed by DNA sequencing with the respective species-specific primers (Table 4, Table S3) followed by phylogenetic analyses using PAUP v.4.0b 10 [65] (Figure S4). The methods used were as in Inderbitzin et al. [4], [47]. Validation of the *V. dahliae – V. longisporum* multiplex PCR assay also included *V. longisporum* lineage A1/D1 strain PD725 from Steventon et al. [44] that was not included in Inderbitzin et al. [47].

### Evaluation of a Previous *V. longisporum* PCR Assay

The presence of the 839-bp intron [43] in *V. longisporum* and *V. dahliae* was assessed with primer pair Bas3 [66] and NS6 [57], with the PCR conditions described above, with an annealing temperature of 48°C and an extension time of 2 min. Isolates screened for the presence of the intron were *V. longisporum* strains PD590 (lineage A1/D1), PD730 (lineage A1/D2), PD614 and PD715 (lineage A1/D3), as well as *V. dahliae* strains Ls.1875 (retrieved as DNA from the Subbarao lab collection) and PD362 [47].

## Supporting Information

Figure S1 ***Verticillium nubilum*** **and** ***V. zaregamsianum*** **PCR assays are species-specific as illustrated by agarose gels of multiplex PCR assays with additional non-target isolates.** Each gel is delimited by 2-log ladders, penultimate wells are negative controls, and relevant size markers are indicated by ‘<’. Lanes are numbered from left to right; numbers are given for every fifth lane. Specificities of PCR assays are given at bottom of gels. For explanation of isolates included see text. S1a. *Verticillium nubilum* PCR assay. Lanes 2, 3: *V. albo-atrum* strains PD670, PD693. Lane 4: *V. alfalfae* strain PD338. Lanes 5–7: *V. dahliae* strains PD322, PD327, PD502. Lanes 8–11: *V. isaacii* strains PD341, PD343, PD618, PD752. Lanes 12, 13: *V. klebahnii* strain PD347, PD407. Lane 14: *V. longisporum* lineage A1/D1 strain PD348. Lane 15: *V. longisporum* lineage A1/D2 strain PD356. Lane 16: *V. longisporum* lineage A1/D3 strain PD589. Lane 17: *V. nonalfalfae* strain PD592. Lanes 18–20: *V. tricorpus* strains PD593, PD685, PD703. Lanes 21–24: *V. zaregamsianum* strains PD740, PD731, PD735, PD739. Lane 25: *Gibellulopsis nigrescens* strain PD710. Lane 26: *Musicillium theobromae* strain PD686. Lane 27: *V. nubilum* strain PD621. Size marker = 1200 bp. S1b. *Verticillium zaregamsianum* PCR assay. Lanes 2, 3: *V. albo-atrum* strains PD670, PD693. Lane 4: *V. alfalfae* strain PD338. Lanes 5–7: *V. dahliae* strains PD322, PD327, PD502. Lanes 8–11: *V. isaacii* strains PD341, PD343, PD618, PD752. Lanes 12, 13: *V. klebahnii* strain PD347, PD407. Lane 14: *V. longisporum* lineage A1/D1 strain PD348. Lane 15: *V. longisporum* lineage A1/D2 strain PD356. Lane 16: *V. longisporum* lineage A1/D3 strain PD589. Lane 17: *V. nonalfalfae* strain PD592. Lane 18: *V. nubilum* strain PD621. Lanes 19–21: *V. tricorpus* strains PD593, PD685, PD703. Lane 22: *Gibellulopsis nigrescens* strain PD710. Lane 23: *Musicillium theobromae* strain PD686. Lane 24: *V. zaregamsianum* strain PD586. Size marker = 300 bp.
(TIF)

Figure S2 **PCR assays correctly identify additional isolates.** Each gel is delimited by 2-log ladders, penultimate wells are negative controls except for Figure S3f, and relevant size markers are indicated by ‘<’. Lanes are numbered from left to right; numbers are given for every fifth lane. Specificities of PCR assays are given at bottom of gels. For explanation of isolates included see text. S2a. *Verticillium dahliae – V. isaacii – V. klebahnii – V. tricorpus* multiplex PCR assay. Lanes 2, 3: *V. isaacii* strains Ls.1868, Ls.1869. Lanes 4–7: *V. dahliae* strains Ls.1871, Ls.1870, Ls.1875, Ls.1878. Lane 8: *V. klebahnii* strain Ls.1886. Lanes 9, 10: *V. dahliae* strains Ls.1877, Ls.1867; Lane 11: *V. klebahnii* strain Ls.1865. Lane 12: *V. isaacii* strain Ls.1864. Size markers = 200, 500 bp. S2b. *Verticillium albo-atrum* PCR assay. Lanes 2–7: *Verticillium albo-atrum* strains PD746, PD747 and PD748, each strain 10 and 100 ng DNA, respectively. Size marker = 700 bp. S2c. *Verticillium alfalfae* PCR assay. Lanes 2–15: *Verticillium alfalfae* strains PD353, PD489, PD681, PD620, PD682, PD683 and PD338, each strain 10 and 100 ng DNA, respectively. Size marker = 1000 bp. S2d. *Verticillium dahliae* PCR assay. Lanes 2–13. *Verticillium dahliae* strains PD323, PD328, PD331, PD615, PD656 and PD718, each strain 10 and 100 ng DNA, respectively. Size marker = 500 bp. S2e. *Verticillium longisporum* PCR assay. Lanes 2–19. *Verticillium longisporum* strains PD640, PD676, PD725, PD402, PD629, PD730, PD589, PD687 and PD715, each strain 10 and 100 ng DNA, respectively. Size markers = 300, 500, 1000 bp. S2f. *Verticillium nonalfalfae* PCR assay. Lanes 2–11. *Verticillium nonalfalfae* strains PD616, PD626, PD744, PD745 and PD808, each strain 10 and 100 ng DNA, respectively. Size marker = 1200 bp. S2g. *Verticillium nonalfalfae* PCR assay. Lanes 2–9. *Verticillium nonalfalfae* strains P809, PD811, PD810 and PD592, each strain 10 and 100 ng DNA, respectively. Size marker = 1200 bp. S2h. *Verticillium nubilum* PCR assay. Lanes 2–9. *Verticillium nubilum* strains PD702, PD741, PD742 and PD621, each strain 10 and 100 ng DNA, respectively. Size marker = 1200 bp. S2i. *Verticillium zaregamsianum* PCR assay. Lanes 2–11. *Verticillium zaregamsianum* strains PD733, PD736, PD737, PD738 and PD740, each strain 10 and 100 ng DNA, respectively. Size marker = 300 bp.
(TIF)

Figure S3 **Multiplex PCR assays are species-specific as illustrated by agarose gels of multiplex PCR assays with non-target isolates.** Each gel is delimited by 2-log ladders; penultimate wells are negative controls, and relevant size markers are indicated by ‘<’. Lanes are numbered from left to right; numbers are given for every fifth lane. Specificities of PCR assays are given at bottom of gels. For explanation of isolates included see text. S3a. *Verticillium albo-atrum – V. alfalfae – V. nonalfalfae* multiplex PCR assay. Lanes 2–4: *V. dahliae* strains PD322, PD327, PD502, respectively. Lanes 5–8: *V. isaacii* strains PD341, PD343, PD618, PD752. Lanes 9, 10: *V. klebahnii* strain PD347, PD407. Lane 11: *V. longisporum* lineage A1/D1 strain PD348. Lane 12: *V. longisporum* lineage A1/D2 strain PD356. Lane 13: *V. longisporum* lineage A1/D3 strain PD589. Lanes 14: *V. nubilum* strain PD621. Lanes 15–17: *V. tricorpus* strains PD593, PD685, PD703. Lanes 18–21: *V. zaregamsianum* strains PD586, PD731, PD735, PD739. Lane 22: *Gibellulopsis nigrescens* strain PD710. Lane 23: *Musicillium theobromae* strain PD686. Lane 24: *V. nonalfalfae* strain PD592. Size marker = 1200 bp. S3b. *Verticillium dahliae – V. isaacii – V. klebahnii – V. tricorpus* multiplex PCR assay. Lanes 2, 3: *V. albo-atrum* strains PD670, PD693. Lane 4: *V. alfalfae* strain PD338. Lane 5: *V. longisporum* lineage A1/D1 strain PD348. Lane 6: *V. longisporum* lineage A1/D2 strain PD356. Lane 7: *V. longisporum* lineage A1/D3 strain PD589. Lane 8: *V. nonalfalfae* strain PD592. Lane 9: *V. nubilum* strain PD621. Lanes 10–13: *V. zaregamsianum* strains PD586, PD731, PD735, PD739. Lane 14: *Gibellulopsis nigrescens* strain PD710. Lane 15: *Musicillium theobromae* strain PD686. Lane 16: *V. dahliae* strain PD363. Size markers = 500 bp. Note that *V. longisporum* lineage A1/D3 has an identical amplicon to *V. dahliae.* S3c. *Verticillium dahliae – V. longisporum* PCR assay. Lanes 2, 3: *V. albo-atrum* strains PD670, PD693. Lane 4: *V. alfalfae* strain PD338. Lanes 5–8: *V. isaacii* strains PD341, PD343, PD618, PD752. Lanes 9, 10: *V. klebahnii* strain PD347, PD407. Lane 11: *V. nonalfalfae* strain PD592. Lane 12: *V. nubilum* strain PD621. Lanes 13–15: *V. tricorpus* strains PD593, PD685, PD703. Lanes 16–19: *V. zaregamsianum* strains PD586, PD731, PD735, PD739. Lane 20: *Gibellulopsis nigrescens* strain PD710. Lane 21: *Musicillium theobromae* strain PD686. Lane 22: *V. dahliae* strain PD678. Size marker = 500 bp. S3d. *Verticillium isaacii – V. klebahnii – V. tricorpus* multiplex PCR assay. Lanes 2, 3: *V. albo-atrum* strains PD670, PD693. Lane 4: *V. alfalfae* strain PD338. Lanes 5–7: *V. dahliae* strains PD322, PD327, PD502. Lane 8: *V. longisporum* lineage A1/D1 strain PD348. Lane 9: *V. longisporum* lineage A1/D2 strain PD356. Lane 10: *V. longisporum* lineage A1/D3 strain PD589. Lane 11: *V. nonalfalfae* strain PD592. Lane 12: *V. nubilum* strain PD621. Lanes 13–16: *V. zaregamsianum* strains PD586, PD731, PD735, PD739. Lane 17: *Gibellulopsis nigrescens* strain PD710. Lane 18: *Musicillium theobromae* strain PD686. Lane 19: *V. isaacii* strain PD341. Size marker = 200 bp.
(TIF)

Figure S4 **Phylogenetic trees confirming the identification of previously genetically uncharacterized strains using the** ***V. dahliae – V. isaacii – V. klebahnii – V. tricorpus*** **multiplex PCR assay.** Shown are most parsimonious trees obtained using representative taxa from Inderbitzin et al. [4] for the *EF* tree on the left, and from Inderbitzin et al. [47] for the ITS tree on the right. See those publications for GenBank accession numbers. Previously unknown strains are in bold and clustered within the species expected based on the multiplex PCR results (Figure S3a).
(TIF)

Table S1 Total counts of positive and negative control isolates used in the validation of *Verticillium* species and *V. longisporum* lineage specific primer pairs in both simplex and multiplex PCR assays.
(DOCX)

Table S2 **GenBank and other accession numbers of DNA sequences used for primer design.**
(DOCX)

Table S3 **Primers designed in this study, primer names reflect deployment in PCR assays:** ‘Aa’ = *V. albo-atrum*, ‘D’ = *V. dahliae* except *V. dahliae* lineage D2, ‘T’ = *V. tricorpus*, ‘A1’ = Species A1, ‘I’ = *V. isaacii*, ‘K’ = *V. klebahnii*, ‘Z’ = *V. zaregamsianum*, ‘Alf’ = *V. alfalfae*, ‘D1’ = Species D1, ‘D3’ = *V. dahliae* lineage D3, ‘No’ = *V. nonalfalfae*, ‘Nu’ = *V. nubilum*; ‘f’ and ‘r’ refer to primer orientation, forward and reverse, respectively.
(DOCX)

Table S4 **Numbers of substitutions at primer sites among a representative sample of** ***Verticillium*** **strains and a** ***Gibellulopsis nigrescens*** **outgroup** [**4]**, [47] **.** Substitution numbers marked by an asterisk are inferred from DNA sequence alignments (Alignments S1, S2, S3, S4, S5), see Table S2 for accession numbers. The remaining substitution numbers are derived from single-locus phylogenetic trees in Inderbitzin et al. [4], [47].
(DOCX)

Alignment S1 **FASTA text file with** ***ACT*** **alignment used for primer design, primer sites are indicated.** Sequence accession numbers are given as part of sequence names for sequences in public databases.
(TXT)

Alignment S2 **FASTA text file with** ***EF*** **alignment used for primer design, primer sites are indicated.** Sequence accession numbers are given as part of sequence names for sequences in public databases.
(TXT)

Alignment S3 **FASTA text file with** ***GPD*** **alignment used for primer design, primer sites are indicated.** Sequence accession numbers are given as part of sequence names for sequences in public databases.
(TXT)

Alignment S4 **FASTA text file with ITS alignment used for primer design, primer sites are indicated.** Sequence accession numbers are given as part of sequence names for sequences in public databases.
(TXT)

Alignment S5 **FASTA text file with** ***TS*** **alignment used for primer design, primer sites are indicated.** Sequence accession numbers are given as part of sequence names for sequences in public databases.
(TXT)
