# Supplementary figures and images for: Identification and Differentiation of Verticillium Species and V. longisporum Lineages by Simplex and Multiplex PCR Assays
Source: PLoS One. 2013 Jun 18;8(6):e65990. doi: 10.1371/journal.pone.0065990 (PMC3688845; doi:10.1371/journal.pone.0065990)

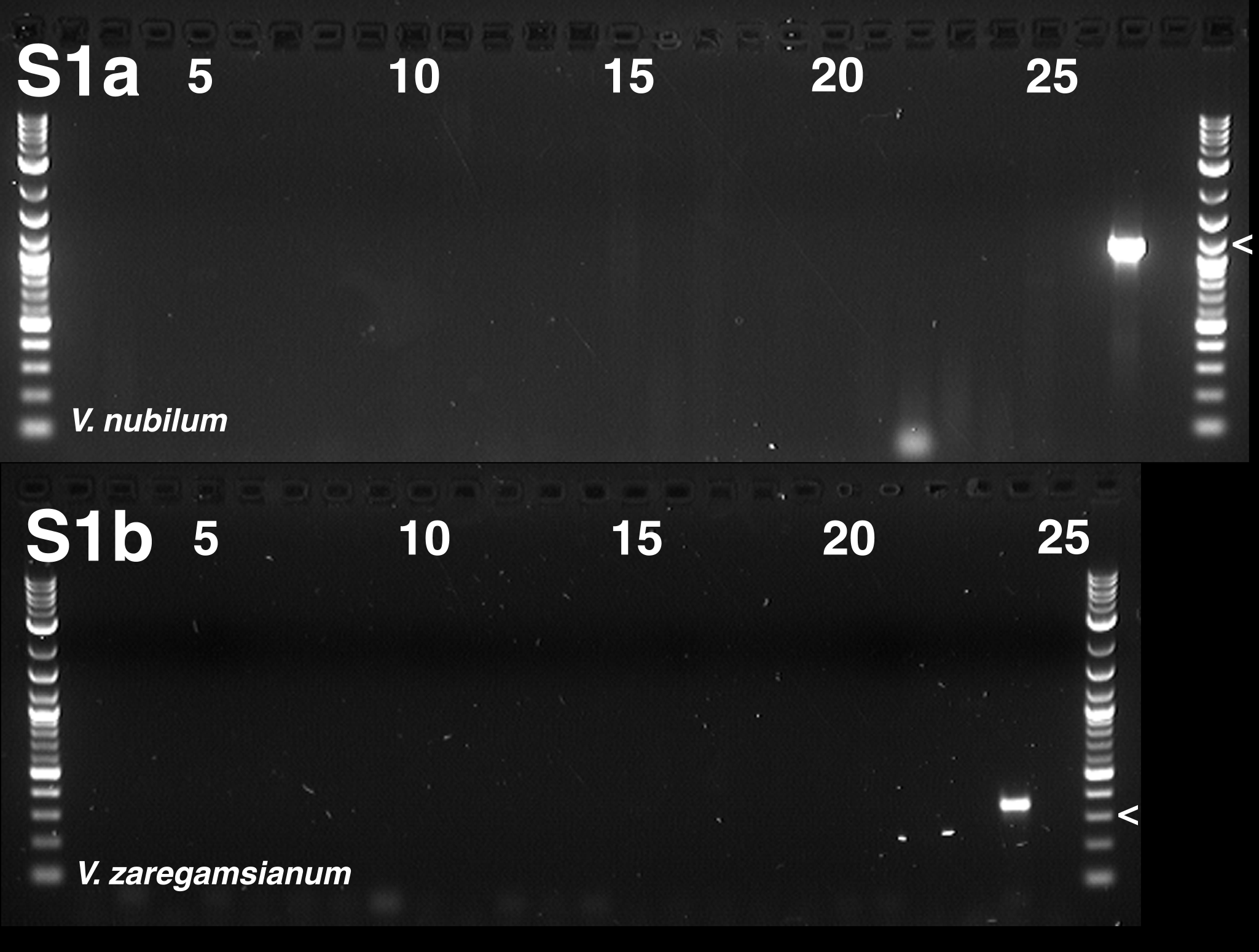

Supplement: Figure S1 — Verticillium nubilum and V. zaregamsianum PCR assays are species-specific as illustrated by agarose gels of multiplex PCR assays with additional non-target isolates. Each gel is delimited by 2-log ladders, penultimate wells are negative controls, and relevant size markers are indicated by ‘<’. Lanes are numbered from left to right; numbers are given for every fifth lane. Specificities of PCR assays are given at bottom of gels. For explanation of isolates included see text. S1a. Verticillium nubilum PCR assay. Lanes 2, 3: V. albo-atrum strains PD670, PD693. Lane 4: V. alfalfae strain PD338. Lanes 5–7: V. dahliae strains PD322, PD327, PD502. Lanes 8–11: V. isaacii strains PD341, PD343, PD618, PD752. Lanes 12, 13: V. klebahnii strain PD347, PD407. Lane 14: V. longisporum lineage A1/D1 strain PD348. Lane 15: V. longisporum lineage A1/D2 strain PD356. Lane 16: V. longisporum lineage A1/D3 strain PD589. Lane 17: V. nonalfalfae strain PD592. Lanes 18–20: V. tricorpus strains PD593, PD685, PD703. Lanes 21–24: V. zaregamsianum strains PD740, PD731, PD735, PD739. Lane 25: Gibellulopsis nigrescens strain PD710. Lane 26: Musicillium theobromae strain PD686. Lane 27: V. nubilum strain PD621. Size marker = 1200 bp. S1b. Verticillium zaregamsianum PCR assay. Lanes 2, 3: V. albo-atrum strains PD670, PD693. Lane 4: V. alfalfae strain PD338. Lanes 5–7: V. dahliae strains PD322, PD327, PD502. Lanes 8–11: V. isaacii strains PD341, PD343, PD618, PD752. Lanes 12, 13: V. klebahnii strain PD347, PD407. Lane 14: V. longisporum lineage A1/D1 strain PD348. Lane 15: V. longisporum lineage A1/D2 strain PD356. Lane 16: V. longisporum lineage A1/D3 strain PD589. Lane 17: V. nonalfalfae strain PD592. Lane 18: V. nubilum strain PD621. Lanes 19–21: V. tricorpus strains PD593, PD685, PD703. Lane 22: Gibellulopsis nigrescens strain PD710. Lane 23: Musicillium theobromae strain PD686. Lane 24: V. zaregamsianum strain PD586. Size marker = 300 bp. (TIF) [file pone.0065990.s001.tif]

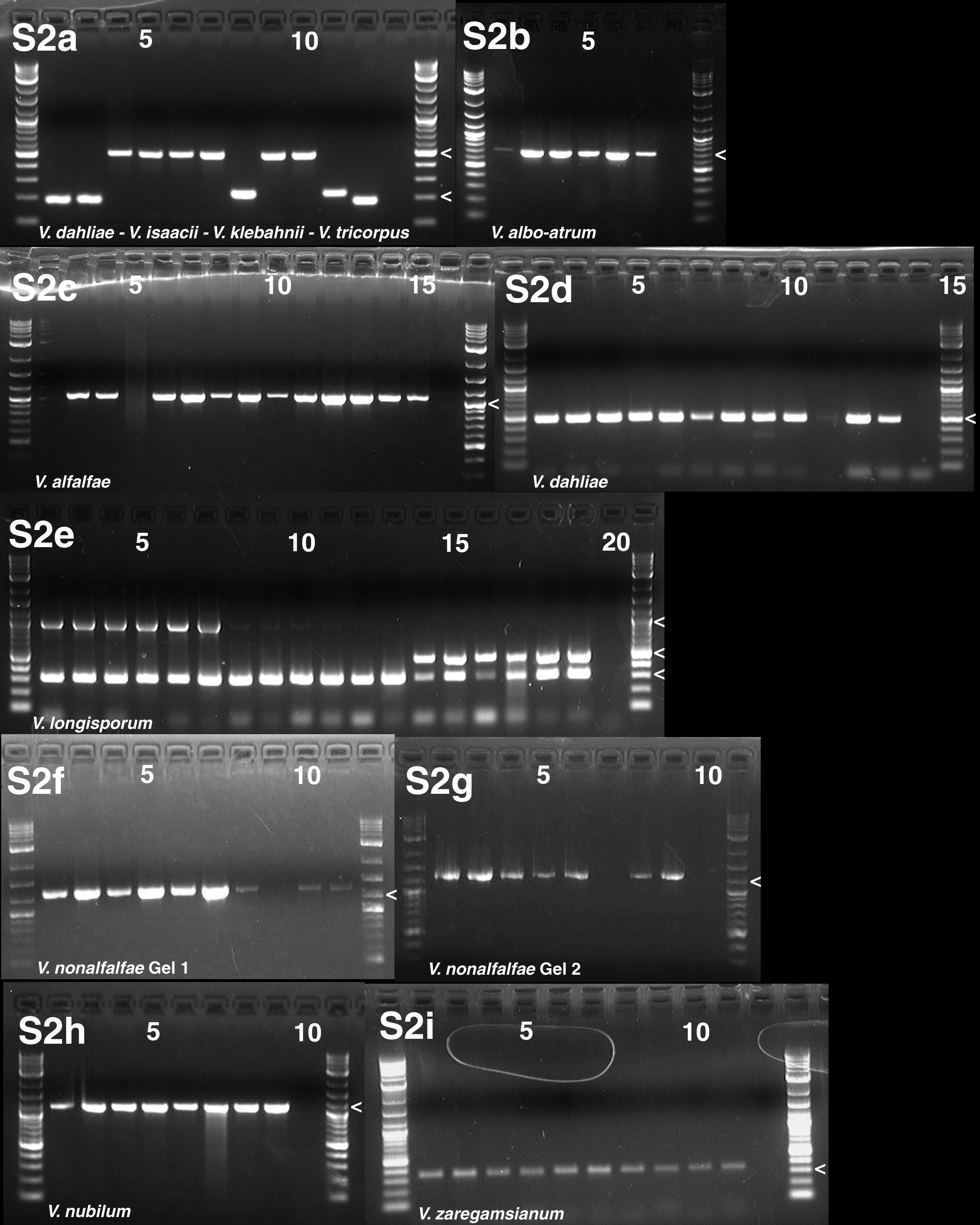

Supplement: Figure S2 — PCR assays correctly identify additional isolates. Each gel is delimited by 2-log ladders, penultimate wells are negative controls except for Figure S3f, and relevant size markers are indicated by ‘<’. Lanes are numbered from left to right; numbers are given for every fifth lane. Specificities of PCR assays are given at bottom of gels. For explanation of isolates included see text. S2a. Verticillium dahliae – V. isaacii – V. klebahnii – V. tricorpus multiplex PCR assay. Lanes 2, 3: V. isaacii strains Ls.1868, Ls.1869. Lanes 4–7: V. dahliae strains Ls.1871, Ls.1870, Ls.1875, Ls.1878. Lane 8: V. klebahnii strain Ls.1886. Lanes 9, 10: V. dahliae strains Ls.1877, Ls.1867; Lane 11: V. klebahnii strain Ls.1865. Lane 12: V. isaacii strain Ls.1864. Size markers = 200, 500 bp. S2b. Verticillium albo-atrum PCR assay. Lanes 2–7: Verticillium albo-atrum strains PD746, PD747 and PD748, each strain 10 and 100 ng DNA, respectively. Size marker = 700 bp. S2c. Verticillium alfalfae PCR assay. Lanes 2–15: Verticillium alfalfae strains PD353, PD489, PD681, PD620, PD682, PD683 and PD338, each strain 10 and 100 ng DNA, respectively. Size marker = 1000 bp. S2d. Verticillium dahliae PCR assay. Lanes 2–13. Verticillium dahliae strains PD323, PD328, PD331, PD615, PD656 and PD718, each strain 10 and 100 ng DNA, respectively. Size marker = 500 bp. S2e. Verticillium longisporum PCR assay. Lanes 2–19. Verticillium longisporum strains PD640, PD676, PD725, PD402, PD629, PD730, PD589, PD687 and PD715, each strain 10 and 100 ng DNA, respectively. Size markers = 300, 500, 1000 bp. S2f. Verticillium nonalfalfae PCR assay. Lanes 2–11. Verticillium nonalfalfae strains PD616, PD626, PD744, PD745 and PD808, each strain 10 and 100 ng DNA, respectively. Size marker = 1200 bp. S2g. Verticillium nonalfalfae PCR assay. Lanes 2–9. Verticillium nonalfalfae strains P809, PD811, PD810 and PD592, each strain 10 and 100 ng DNA, respectively. Size marker = 1200 bp. S2h. Verticillium nubilum PCR assay. Lanes 2–9. Ver [file pone.0065990.s002.tif]

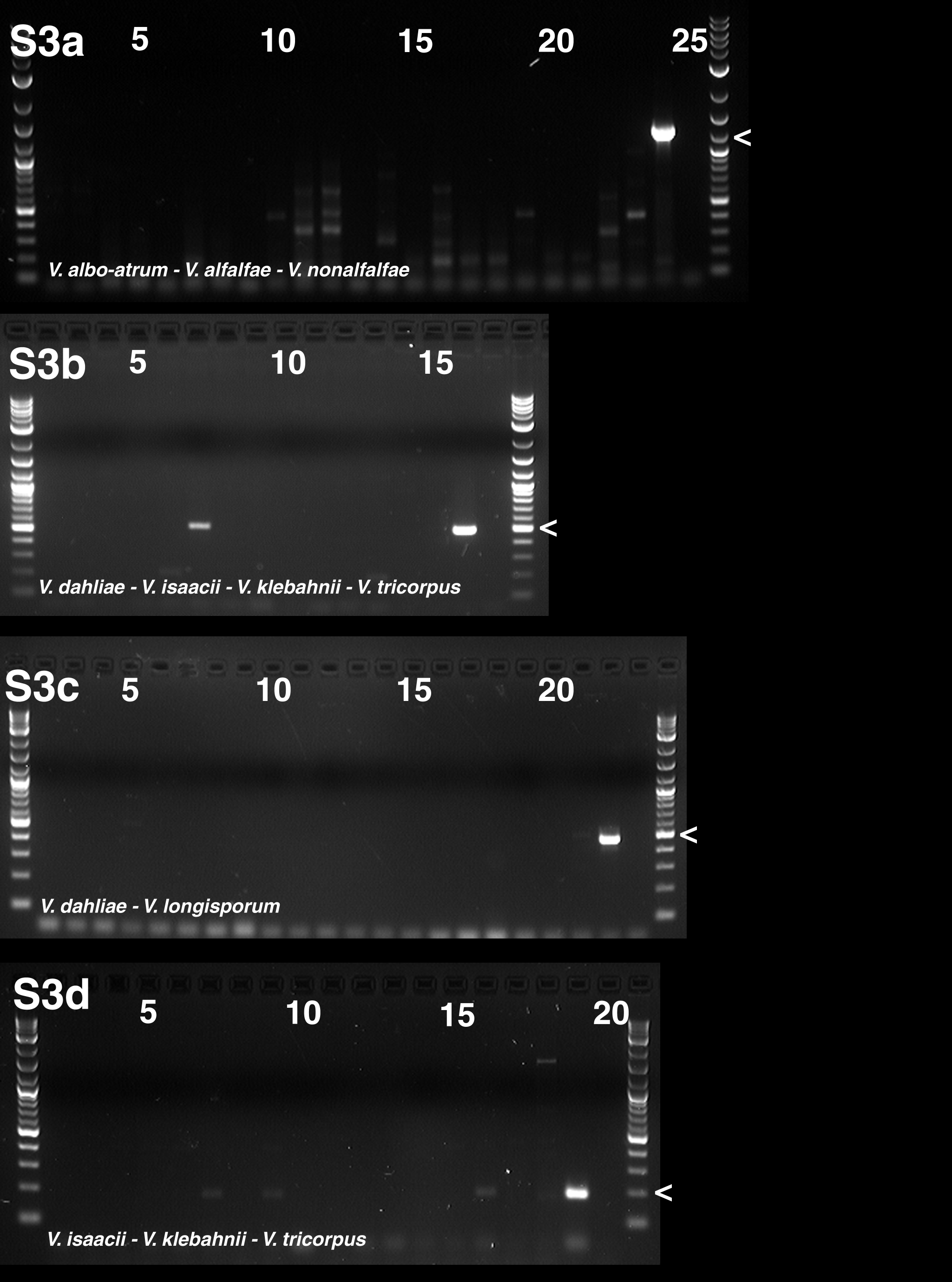

Supplement: Figure S3 — Multiplex PCR assays are species-specific as illustrated by agarose gels of multiplex PCR assays with non-target isolates. Each gel is delimited by 2-log ladders; penultimate wells are negative controls, and relevant size markers are indicated by ‘<’. Lanes are numbered from left to right; numbers are given for every fifth lane. Specificities of PCR assays are given at bottom of gels. For explanation of isolates included see text. S3a. Verticillium albo-atrum – V. alfalfae – V. nonalfalfae multiplex PCR assay. Lanes 2–4: V. dahliae strains PD322, PD327, PD502, respectively. Lanes 5–8: V. isaacii strains PD341, PD343, PD618, PD752. Lanes 9, 10: V. klebahnii strain PD347, PD407. Lane 11: V. longisporum lineage A1/D1 strain PD348. Lane 12: V. longisporum lineage A1/D2 strain PD356. Lane 13: V. longisporum lineage A1/D3 strain PD589. Lanes 14: V. nubilum strain PD621. Lanes 15–17: V. tricorpus strains PD593, PD685, PD703. Lanes 18–21: V. zaregamsianum strains PD586, PD731, PD735, PD739. Lane 22: Gibellulopsis nigrescens strain PD710. Lane 23: Musicillium theobromae strain PD686. Lane 24: V. nonalfalfae strain PD592. Size marker = 1200 bp. S3b. Verticillium dahliae – V. isaacii – V. klebahnii – V. tricorpus multiplex PCR assay. Lanes 2, 3: V. albo-atrum strains PD670, PD693. Lane 4: V. alfalfae strain PD338. Lane 5: V. longisporum lineage A1/D1 strain PD348. Lane 6: V. longisporum lineage A1/D2 strain PD356. Lane 7: V. longisporum lineage A1/D3 strain PD589. Lane 8: V. nonalfalfae strain PD592. Lane 9: V. nubilum strain PD621. Lanes 10–13: V. zaregamsianum strains PD586, PD731, PD735, PD739. Lane 14: Gibellulopsis nigrescens strain PD710. Lane 15: Musicillium theobromae strain PD686. Lane 16: V. dahliae strain PD363. Size markers = 500 bp. Note that V. longisporum lineage A1/D3 has an identical amplicon to V. dahliae. S3c. Verticillium dahliae – V. longisporum PCR assay. Lanes 2, 3: V. albo-atrum strains PD670, PD693. Lane 4: V. alfalfae strain PD338. Lanes 5–8: V. isaac [file pone.0065990.s003.tif]

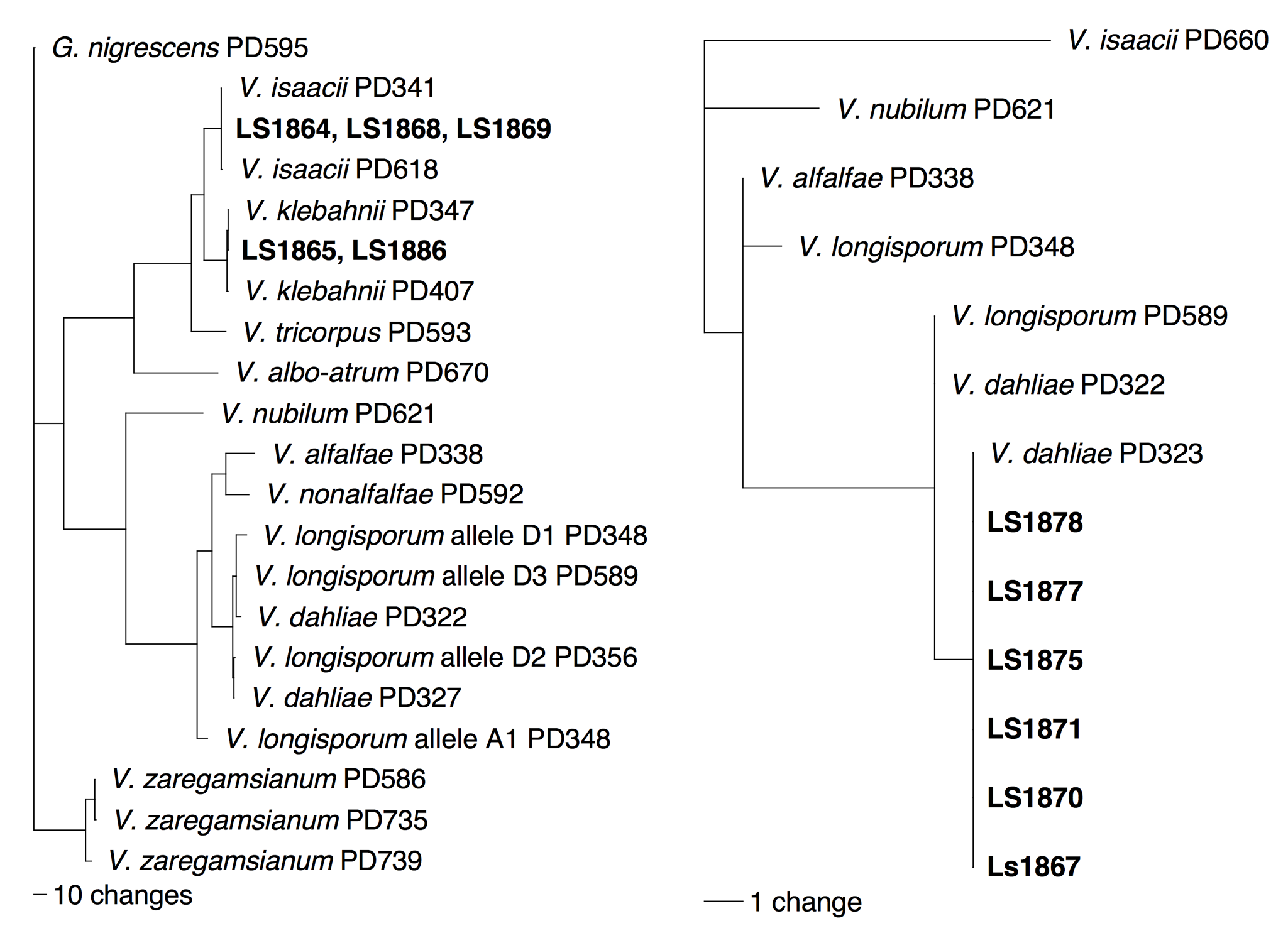

Supplement: Figure S4 — Phylogenetic trees confirming the identification of previously genetically uncharacterized strains using the V. dahliae – V. isaacii – V. klebahnii – V. tricorpus multiplex PCR assay. Shown are most parsimonious trees obtained using representative taxa from Inderbitzin et al. [4] for the EF tree on the left, and from Inderbitzin et al. [47] for the ITS tree on the right. See those publications for GenBank accession numbers. Previously unknown strains are in bold and clustered within the species expected based on the multiplex PCR results (Figure S3a). (TIF) [file pone.0065990.s004.tif]
