# Supplementary material for: Identification and Differentiation of Verticillium Species and V. longisporum Lineages by Simplex and Multiplex PCR Assays
Source: PLoS One. 2013 Jun 18;8(6):e65990. doi: 10.1371/journal.pone.0065990 (PMC3688845; doi:10.1371/journal.pone.0065990)
Supplement: Table S1 — Total counts of positive and negative control isolates used in the validation of Verticillium species and V. longisporum lineage specific primer pairs in both simplex and multiplex PCR assays. (DOCX) [file pone.0065990.s005.docx]

Table S1. Total counts of positive and negative control isolates used in the validation of *Verticillium* species and *V. longisporum* lineage specific primer pairs in both simplex and multiplex PCR assays.

| Species | Total target isolates^A^ | Total negative controls^B^ |
| --- | --- | --- |
| *V. albo-atrum* | 5 | 24 |
| *V. alfalfae* | 7 | 25 |
| *V. dahliae* including *V. longisporum*  allele D3 | 16 | 27 |
| *V. isaacii* | 7 | 30 |
| *V. klebahnii* | 4 | 33 |
| *V. nonalfalfae* | 8 | 25 |
| *V. nubilum* | 3 | 25 |
| *V. tricorpus* | 3 | 24 |
| *V. zaregamsianum* | 9 | 22 |
| Species A1 | 12 | 25 |
| Species D1 | 5 | 33 |

^A^ Unique target isolates across Table 3, Table 5 and Figure S3 for *V. dahliae, V. isaacii, V. klebahnii, V. tricorpus*, Species A1 and Species D1.

^B^ Unique negative control isolates across Table 3, Table 5 and Figure S3 for *V. dahliae, V. isaacii, V. klebahnii* and *V. tricorpus*, Species A1 and Species D1.
