# Supplementary material for: Identification and Differentiation of Verticillium Species and V. longisporum Lineages by Simplex and Multiplex PCR Assays
Source: PLoS One. 2013 Jun 18;8(6):e65990. doi: 10.1371/journal.pone.0065990 (PMC3688845; doi:10.1371/journal.pone.0065990)
Supplement: Table S2 — GenBank and other accession numbers of DNA sequences used for primer design. (DOCX) [file pone.0065990.s006.docx]

Table S2. GenBank and other accession numbers of DNA sequences used for primer design.

| **Species** | **Strain** | ***ACT*** | ***EF*** | ***GPD*** | **ITS** | ***TS*** |
| --- | --- | --- | --- | --- | --- | --- |
| *G. nigrescens* | PD595 | JN188102 | JN188230 | JN188166 | JN187976 | JN188038 |
| *V. albo-atrum* | PD670 | JN188116 | JN188244 | JN188180 | JN187990 | JN188052 |
| *V. albo-atrum* | PD693 | JN188122 | - | - | - | - |
| *V. alfalfae* | PD338 | JN188088 | JN188216 | JN188152 | HQ206733 | JN188024 |
| *V. alfalfae* | PD683 | - | - | - | JN187991 | - |
| *V. dahliae* | PD322 | HQ206921 | HQ414624 | HQ414719 | HQ206718 | HQ414909 |
| *V. dahliae* | PD323 | HQ206922 | HQ414625 | HQ414720 | HQ206719 | - |
| *V. dahliae* | PD327 | - | HQ414628 | HQ414723 | - | HQ414913 |
| *V. dahliae* | PD331 | - | - | - | - | HQ414915 |
| *V. dahliae* | PD332 | - | HQ414631 | - | - | - |
| *V. dahliae* | PD502 | - | - | - | - | HQ414930 |
| *V. dahliae* | PD585 | - | - | HQ414748 | - | - |
| *V. dahliae* | PD656 | - | - | - | - | HQ414972 |
| *V. dahliae* | Vd.Ls17 | VDAG_08445^A^ | - | VDAG_08916^A^ | - | VDAG_01254^A^ |
| *V. dahliae* | 76 Greece | - | - | - | AF104926 | - |
| *V. isaacii* | PD341 | JN188089 | JN188217 | JN188153 | JN187963 | JN188025 |
| *V. isaacii* | PD343 | - | - | - | - | JN188026 |
| *V. isaacii* | PD618 | - | JN188237 | JN188173 | - | JN188045 |
| *V. isaacii* | PD752 | - | - | - | - | JN188082 |
| *V. klebahnii* | PD347 | JN188091 | JN188219 | JN188155 | JN187965 | JN188027 |
| *V. klebahnii* | PD407 | - | JN188222 | JN188158 | JN187968 | JN188030 |
| *V. longisporum* allele A1 | PD348 | **-** | HQ414633 | - | HQ206738 | - |
| *V. longisporum* allele A1 | PD356 | HQ206934 | - | - | - | - |
| *V. longisporum* allele A1 | PD588 | - | - | HQ414750 | - | HQ414940 |
| *V. longisporum* allele D1 | PD348 | - | HQ414634 | - | - | - |
| *V. longisporum* allele D1 | PD588 | - | - | HQ414751 | - | HQ414941 |
| *V. longisporum* allele D1 | PD591 | HQ206959 | - | - | - | - |
| *V. longisporum* allele D2 | PD356 | HQ206935 | HQ414638 | HQ414733 | - | - |
| *V. longisporum* allele D2 | PD402 | - | - | - | - | HQ414927 |
| *V. longisporum* allele D3 | PD589 | - | - | - | HQ206832 | HQ414943 |
| *V. longisporum* allele D3 | PD614 | - | HQ414665 | - | - | - |
| *V. nonalfalfae* | PD592 | JN188099 | JN188227 | JN188163 | JN187973 | JN188035 |
| *V. nubilum* | PD621 | HQ206966 | HQ414669 | HQ414764 | HQ206852 | HQ414954 |
| *V. tricorpus* | PD593 | JN188100 | JN188228 | JN188164 | JN187974 | JN188036 |
| *V. tricorpus* | PD685 | - | - | JN188184 | - | - |
| *V. tricorpus* | PD703 | - | - | JN188188 | - | - |
| *V. zaregamsianum* | PD586 | JN188098 | JN188226 | JN188162 | JN187972 | JN188034 |
| *V. zaregamsianum* | PD731 | - | - | JN188193 | - | JN188065 |
| *V. zaregamsianum* | PD735 | - | JN188260 | - | - | JN188068 |
| *V. zaregamsianum* | PD739 | JN188136 | JN188264 | JN188200 | JN188008 | JN188072 |

^A^Sequence retrieved from the Broad Institute website (http://www.broadinstitute.org/annotation​/genome/verticillium_dahliae/MultiHome.h​tml, accessed February 10, 2009).
