# Supplementary material for: Identification and Differentiation of Verticillium Species and V. longisporum Lineages by Simplex and Multiplex PCR Assays
Source: PLoS One. 2013 Jun 18;8(6):e65990. doi: 10.1371/journal.pone.0065990 (PMC3688845; doi:10.1371/journal.pone.0065990)
Supplement: Table S3 — Primers designed in this study, primer names reflect deployment in PCR assays: ‘Aa’ = V. albo-atrum, ‘D’ = V. dahliae except V. dahliae lineage D2, ‘T’ = V. tricorpus, ‘A1’ = Species A1, ‘I’ = V. isaacii, ‘K’ = V. klebahnii, ‘Z’ = V. zaregamsianum, ‘Alf’ = V. alfalfae, ‘D1’ = Species D1, ‘D3’ = V. dahliae lineage D3, ‘No’ = V. nonalfalfae, ‘Nu’ = V. nubilum; ‘f’ and ‘r’ refer to primer orientation, forward and reverse, respectively. (DOCX) [file pone.0065990.s007.docx]

Table S3. Primers designed in this study, given are primer names, target loci, primer sequences and the primer melting temperatures, primer names reflect deployment in PCR assays: 'Aa' = *V. albo-atrum*, 'D' = *V. dahliae* and *V. longisporum* lineage A1/D3, 'T' = *V. tricorpus*, 'A1' = Species A1, 'I' = *V. isaacii*, 'K' = *V. klebahnii*, 'Z' = *V. zaregamsianum*, 'Alf' = *V. alfalfae*, 'D1' = Species D1, 'No' = *V. nonalfalfae*, 'Nu' = *V. nubilum*; 'f' and 'r' refer to primer orientation, forward and reverse, respectively.

| **Primer name** | **Locus** | **Sequence (5' -> 3')** | **Melting temperature, °C** |
| --- | --- | --- | --- |
| AaTr | *ACT* | CTGGATGGAGACGTAGAAGGC | 55 |
| AaF | *ACT* | GGCCTCGATAGCATCGCC | 58 |
| Tf | *ACT* | CGTGCTGTCTTCCGTAAGTTTG | 56 |
| A1f | *EF* | AAGTGGAGCCCCGTATCTTGAAT | 56 |
| A1r | *EF* | CAACTGGCAACAGGGCTTGAAT | 56 |
| If | *EF* | CGATGTCGCGATGACCTCG | 57 |
| IKr | *EF* | CGGCAGCCTCCTAAACATGG | 56 |
| Kf | *EF* | ACATCCTGAGGCTGCTTGAGA | 56 |
| AlfD1r | *GPD* | TGCCGGCATCGACCTTGG | 56 |
| Alff | *GPD* | TCATGCCCCCTTTGTTCATCGAT | 57 |
| D1f | *GPD* | CCCCGGCCTTGGTCTGAT | 54 |
| Zf | *GPD* | GGTTTCCTCCCCTCACACG | 53 |
| Zr | *GPD* | CCACCCTTGATGTGGGCGGA | 57 |
| Df | ITS | CCGGTCCATCAGTCTCTCTG | 54 |
| Dr | ITS | CTGTTGCCGCTTCACTCG | 55 |
| NoF | *TS* | CCTCGAAAAATCCACCAGCTCTA | 56 |
| NoNuR | *TS* | GTGGTTGAGATCCTCACGCTTC | 56 |
| Nuf | *TS* | GGTCCCCCTCGTTCATGCAATC | 56 |
