# Supplementary material for: Identification and Differentiation of Verticillium Species and V. longisporum Lineages by Simplex and Multiplex PCR Assays
Source: PLoS One. 2013 Jun 18;8(6):e65990. doi: 10.1371/journal.pone.0065990 (PMC3688845; doi:10.1371/journal.pone.0065990)
Supplement: Table S4 — Numbers of substitutions at primer sites among a representative sample of Verticillium strains and a Gibellulopsis nigrescens outgroup [4] , [47] . Substitution numbers marked by an asterisk are inferred from DNA sequence alignments (Alignments S1, S2, S3, S4, S5), see Table S2 for accession numbers. The remaining substitution numbers are derived from single-locus phylogenetic trees in Inderbitzin et al. [4], [47]. (DOCX) [file pone.0065990.s008.docx]

Table S4. Numbers of substitutions at primer sites among a representative sample of *Verticillium* strains and a *Gibellulopsis nigrescens* outgroup [[1](#_ENREF_1),[2](#_ENREF_2)]. Numbers marked by an asterisk are the numbers of substitutions between homologous primer sites inferred from DNA sequence alignments (Alignment S1, S2, S3, S4, S5), see Table S2 for accession numbers. The remaining numbers of substitutions are derived from the respective single-locus phylogenetic trees in Inderbitzin et al. [[1](#_ENREF_1),[2](#_ENREF_2)].

| **Locus** | ***ACT*** | | | ***EF*** | | | | | ***GPD*** | | | | | ***TS*** | | | **ITS** | |
| --- | --- | --- | --- | --- | --- | --- | --- | --- | --- | --- | --- | --- | --- | --- | --- | --- | --- | --- |
| Primer (length, bp) | AaF (18) | AaDTr (21)^A^ | Tf (22) | A1f (23) | A1r (22) | If (19) | IKr (20) | Kf (21) | AlfF (23) | AlfD1r (18)^B^ | D1f (18) | Zf (19) | Zr (20) | NoF (23) | NoNuR (22)^C^ | NuF (22) | D3f (20) | D3r (18)^D^ |
| *V. albo-atrum* PD670 | 1* | n.d.^E^ | 2* | 17* | n.a.*^F^ | 18* | 9* | 8* | 14* | n.d. | 13* | 10* | 0* | 19 | n.d. | 14* | 3* | n.d. |
| *V. albo-atrum* PD693 | 0* | n.d. | 2* | 17 | n.a. | 18 | 9 | 8 | 14 | n.d. | 13 | 10 | 0 | 19 | n.d. | 14 | 3 | n.d. |
| *V. alfalfae* PD338 | 7* | n.d. | 2* | 7* | 3* | 15* | 9* | 14* | 0* | n.d. | 7* | 13* | 3* | 2 | n.d. | 16* | 3* | n.d. |
| *V. alfalfae* PD683 | 7 | n.d. | 2 | 7 | 3 | 15 | 9 | 14 | 0 | n.d. | 7 | 13 | 3 | 2 | n.d. | 16 | 3* | n.d. |
| *V. dahliae* PD322 | 7* | 0 | 2* | 4* | 3* | 14* | 10* | 13* | 18* | 0 | 2* | 14* | 3* | 7 | 0 | 16* | 0* | n.d. |
| *V. dahliae* PD323 | 7* | n.d. | 2* | 4* | 3* | 14* | 10* | 14* | 18* | n.d. | 2* | 14* | 3* | 7 | n.d. | 16 | 0* | n.d. |
| *V. dahliae* PD327 | 7 | n.d. | 2 | 4* | 3* | 14* | 10* | 14* | 18* | n.d. | 2* | 14* | 3* | 7 | n.d. | 16* | 0 | n.d. |
| *V. dahliae* PD331 | 7 | n.d. | 2 | 4 | 3 | 14 | 10 | 14 | 18 | n.d. | 2 | 14 | 3 | 7 | n.d. | 16* | 0 | n.d. |
| *V. dahliae* PD332 | 7 | n.d. | 2 | 4* | 3* | 14* | 10* | 14* | 18 | n.d. | 2 | 14 | 3 | 7 | n.d. | 16 | 0 | n.d. |
| *V. dahliae* PD502 | 7 | n.d. | 2 | 4 | 3 | 14 | 10 | 14 | 18 | n.d. | 2 | 14 | 3 | 7 | n.d. | 16* | 0 | n.d. |
| *V. dahliae* PD585 | 7 | n.d. | 2 | 4 | 3 | 14 | 10 | 14 | 18* | n.d. | 2* | 14* | 3* | 7 | n.d. | 16 | 0 | n.d. |
| *V. dahliae* PD656 | 7 | n.d. | 2 | 4 | 3 | 14 | 10 | 14 | 18 | n.d. | 2 | 14 | 3 | 7 | n.d. | 16* | 0 | n.d. |
| *V. isaacii* PD341 | 6* | n.d. | 2* | 18* | n.a.* | 0* | 0* | 7* | 19* | n.d. | 10* | 6* | 0* | 18 | n.d. | 18* | 3* | n.d. |
| *V. isaacii* PD343 | 6 | n.d. | 2 | 18 | n.a. | 0 | 0 | 7 | 19 | n.d. | 10 | 6 | 0 | 18 | n.d. | 18* | 3 | n.d. |
| *V. isaacii* PD618 | 6 | n.d. | 2 | 18* | n.a.* | 0* | 0* | 7* | 19* | n.d. | 10* | 6* | 0* | 18 | n.d. | 18* | 3 | n.d. |
| *V. isaacii* PD752 | 6 | n.d. | 2 | 18 | n.a. | 0 | 0 | 7 | 19 | n.d. | 10 | 6 | 0 | 18 | n.d. | 18* | 3 | n.d. |
| *V. klebahnii* PD347 | 6* | n.d. | 2* | 18* | n.a.* | 2* | 0* | 0* | 18* | n.d. | 10* | 5* | 0* | 18 | n.d. | 15* | 3* | n.d. |
| *V. klebahnii* PD407 | 6 | n.d. | 2 | 18* | n.a.* | 2* | 0* | 0* | 18* | n.d. | 10* | 5* | 0* | 18 | n.d. | 15* | 3* | n.d. |
| *V. longisporum* allele A1 PD348 | 7 | n.d. | 1 | 0* | 0* | 14* | 10* | 13* | 15 | n.d. | 2 | 12 | 3 | 6 | n.d. | 15 | 3* | n.d. |
| *V. longisporum* allele A1 PD356 | 7* | n.d. | 1* | 0 | 0 | 14 | 10 | 13 | 15 | n.d. | 2 | 12 | 3 | 6 | n.d. | 15 | 3 | n.d. |
| *V. longisporum* allele A1 PD588 | 7 | n.d. | 1 | 0 | 0 | 14 | 10 | 13 | 15* | n.d. | 2* | 12* | 3* | 6 | n.d. | 15* | 3 | n.d. |
| *V. longisporum* allele D1 PD348 | 6 | n.d. | 3 | 5* | 3* | 14* | 10* | 13* | 16 | n.d. | 0 | 13 | 3 | 8 | n.d. | 16 | 3 | n.d. |
| *V. longisporum* allele D1 PD588 | 6 | n.d. | 3 | 5 | 3 | 14 | 10 | 13 | 16* | n.d. | 0* | 13* | 3* | 8 | n.d. | 16* | 3 | n.d. |
| *V. longisporum* allele D1 PD591 | 6* | n.d. | 3* | 5 | 3 | 14 | 10 | 13 | 16 | n.d. | 0 | 13 | 3 | 8 | n.d. | 16 | 3 | n.d. |
| *V. longisporum* allele D2 PD356 | 7* | n.d. | 2* | 3* | 3* | 14* | 10* | 14* | 17* | n.d. | 2* | 14* | 3* | 7 | n.d. | 16 | 3 | n.d. |
| *V. longisporum* allele D2 PD402 | 7 | n.d. | 2 | 3 | 3 | 14 | 10 | 14 | 17 | n.d. | 2 | 14 | 3 | 7 | n.d. | 16* | 3 | n.d. |
| *V. longisporum* allele D3 PD589 | 7 | n.d. | 2 | 3 | 3 | 14 | 10 | 14 | 17 | n.d. | 2 | 14 | 3 | 7 | n.d. | 16* | 0* | n.d. |
| *V. longisporum* allele D3 PD614 | 7 | n.d. | 2 | 3* | 3* | 14* | 10* | 13* | 17 | n.d. | 2 | 14 | 3 | 7 | n.d. | 16 | 0 | n.d. |
| *V. nonalfalfae* PD592 | 9* | n.d. | 2* | 8* | 3* | 15* | 10* | 13* | 4* | n.d. | 7* | 13* | 3* | 0 | n.d. | 15* | 3* | n.d. |
| *V. nubilum* PD621 | 6* | n.d. | 2* | 14* | 12* | 16* | 10* | 16* | 20* | n.d. | 16* | 12* | 3* | 15 | n.d. | 0* | 3* | n.d. |
| *V. tricorpus* PD593 | 6* | n.d. | 0* | 17* | n.a.* | 4* | 1* | 8* | 18* | n.d. | 11* | 3* | 0* | 17 | n.d. | 13* | 3* | n.d. |
| *V. tricorpus* PD685 | 6 | n.d. | 0 | 17 | n.a. | 4 | 1 | 8 | 18* | n.d. | 11* | 3* | 0* | 17 | n.d. | 13 | 3 | n.d. |
| *V. tricorpus* PD703 | 6 | n.d. | 0 | 17 | n.a. | 4 | 1 | 8 | 18* | n.d. | 11* | 3* | 0* | 17 | n.d. | 13 | 3 | n.d. |
| *V. zaregamsianum* PD586 | 7* | n.d. | 2* | 15* | n.a. | 16* | 9* | 11* | 15* | n.d. | 17* | 0* | 0* | 18 | n.d. | 14* | 3* | n.d. |
| *V. zaregamsianum* PD731 | 7 | n.d. | 2 | 15 | n.a. | 16 | 9 | 11 | 15* | n.d. | 17* | 0* | 0* | 18 | n.d. | 14* | 3 | n.d. |
| *V. zaregamsianum* PD735 | 7 | n.d. | 2 | 15* | n.a.* | 16* | 9* | 11* | 15 | n.d. | 17 | 0 | 0 | 18 | n.d. | 14* | 3 | n.d. |
| *V. zaregamsianum* PD739 | 6* | n.d. | 1* | 15* | n.a.* | 16* | 8* | 10* | 17* | n.d. | 17* | 1* | 0* | 18 | n.d. | 14* | 3* | n.d. |
| *G. nigrescens* PD595 | 11* | n.d. | 4* | 16* | n.a.* | n.a.* | n.a.* | n.a.* | 18* | n.d. | 14* | 10* | 9* | 18 | n.d. | 13* | 7* | n.d. |

^A^AaDTr was designed based on *V. dahliae* strain PD322 (=VdLs.17) *ACT* coding region (Accession: VDAG_08445) [[3](#_ENREF_3)]. Primer sites in remaining isolates not determined.

^B^AlfD1r was designed based on *V. dahliae* strain PD322 (=VdLs.17) *GPD* coding region (Accession: VDAG_08916) [[3](#_ENREF_3)]. Primer sites in remaining isolates not determined.

^C^NoNuR was designed based on *V. dahliae* strain PD322 (=VdLs.17) *TS* coding region (Accession: VDAG_01254) [[3](#_ENREF_3)]. Primer sites in remaining isolates not determined.

^D^D3r was designed based on a *V. dahliae* 28S sequence (Accession: AF104926) [[4](#_ENREF_4)]. Primer sites in isolates used in this study not determined.

^E^n.d. = Not determined.

^F^n.a. = Not alignable (site missing).

^G^Seven different *V. longisporum* isolates were used for primer design across all loci, because sequencing coverage differed for isolates between loci. The genetic diversity within the *V. longisporum* isolates in Inderbitzin et al. [[2](#_ENREF_2)] across the five loci used in this study can be represented by three isolates.

*DNA sequence of this isolate was used for primer design and was included in Alignment S1, S2, S3, S4 or S5.

References:

1. Inderbitzin P, Bostock RM, Davis RM, Usami T, Platt HW, et al. (2011) Phylogenetics and taxonomy of the fungal vascular wilt pathogen *Verticillium*, with the descriptions of five new species. PLoS ONE 6: e28341.

2. Inderbitzin P, Davis RM, Bostock RM, Subbarao KV (2011) The ascomycete *Verticillium longisporum* is a hybrid and a plant pathogen with an expanded host range. PLoS ONE 6: e18260.

3. Klosterman SJ, Subbarao KV, Kang S, Veronese P, Gold SE, et al. (2011) Comparative genomics yields insights into niche adaptation of plant vascular wilt pathogens. PLoS Pathog 7: e1002137.

4. Pramateftaki PV, Antoniou PP, Typas MA (2000) The complete DNA sequence of the nuclear ribosomal RNA gene complex of *Verticillium dahliae*: Intraspecific heterogeneity within the intergenic spacer region. Fungal Genet Biol 29: 19-27.
